# Long term transcriptional and behavioral effects in mice developmentally exposed to a mixture of endocrine disruptors associated with delayed human neurodevelopment

**DOI:** 10.1038/s41598-020-66379-x

**Published:** 2020-06-09

**Authors:** Anastasia Repouskou, Anastasia-Konstantina Papadopoulou, Emily Panagiotidou, Panagiotis Trichas, Christian Lindh, Åke Bergman, Chris Gennings, Carl-Gustaf Bornehag, Joëlle Rüegg, Efthymia Kitraki, Antonios Stamatakis

**Affiliations:** 1 Basic Sciences lab, Faculty of Dentistry, School of Health Sciences, National and Kapodistrian University of Athens (NKUA), Athens, Greece; 2 Biology-Biochemistry lab, Faculty of Nursing, School of Health Sciences, NKUA, Athens, Greece; 3 Division of Occupational and Environmental Medicine, Department of Laboratory Medicine, Lund University, Lund, Sweden; 4 Department of Environmental Science, Stockholm University, SE-106 91, Stockholm, Sweden; 5 Icahn School of Medicine at Mount Sinai, New York, NY USA; 6 Karlstad University, Karlstad, Sweden; 7 Uppsala University, Evolutionary Biology Centre, Department of Organismal Biology 18 A, Norbyvägen, 752 36, Uppsala, Sweden

**Keywords:** Stress and resilience, Neuroscience, Endocrinology

## Abstract

Accumulating evidence suggests that gestational exposure to endocrine disrupting chemicals (EDCs) may interfere with normal brain development and predispose for later dysfunctions. The current study focuses on the exposure impact of mixtures of EDCs that better mimics the real-life situation. We herein describe a mixture of phthalates, pesticides and bisphenol A (mixture N1) detected in pregnant women of the SELMA cohort and associated with language delay in their children. To study the long-term impact of developmental exposure to N1 on brain physiology and behavior we administered this mixture to mice throughout gestation at doses 0×, 0.5×, 10×, 100× and 500× the geometric mean of SELMA mothers’ concentrations, and examined their offspring in adulthood. Mixture N1 exposure increased active coping during swimming stress in both sexes, increased locomotion and reduced social interaction in male progeny. The expression of corticosterone receptors, their regulator *Fkbp5*, corticotropin releasing hormone and its receptor, oxytocin and its receptor, estrogen receptor beta, serotonin receptors (*Htr1a, Htr2a*) and glutamate receptor subunit *Grin2b*, were modified in the limbic system of adult animals, in a region-specific, sexually-dimorphic and experience-dependent manner. Principal component analysis revealed gene clusters associated with the observed behavioral responses, mostly related to the stress axis. This integration of epidemiology-based data with an experimental model increases the evidence that prenatal exposure to EDC mixtures impacts later life brain functions.

## Introduction

Endocrine disrupting chemicals (EDCs) can interfere with the physiological actions of hormones, resulting in abnormal endocrine functioning^1,2^. Developing organisms are more susceptible to EDCs due to their immature detoxifying systems and the crucial organizational role of hormones in the development of many organs^1^. The developing nervous system is significantly impacted by different categories of EDCs including phthalates, phenols and pesticides that disrupt the normal pattern of hormonal actions and neurotransmission^3,4^. In humans, early exposures to these chemicals have been associated with childhood hyperactivity and are considered possible contributors to the emergence of Attention Deficit Hyperactivity Disorder (ADHD) and Autism Spectrum Disorder (ASD)^5–9^. Likewise, in animal studies exposures during development to phthalates^10^, pesticides^11^ or bisphenol A (BPA)^12^ can impair hormonal and neurotransmission homeostasis, locomotor activity, emotionality and cognitive function, leading to lifelong or even transgenerational dysfunctions^13^.

To date, the list of identified EDCs includes approximately 1000 different chemicals, to which humans and wildlife are exposed through a variety of sources including plastics, pesticides, personal care products, and flame retardants. The prevalence of EDCs in the environment and inside the organisms, as well as the plethora of affected hormonal systems have placed these chemicals in the list of worldwide health challenges^14^. However, while most studies test one chemical at the time, in real life organisms are exposed to many EDCs simultaneously. In mixtures, EDCs might have additive, synergistic or opposing effects, as shown by previous studies examining the impact of single EDCs and their mixtures. While a number of studies have addressed mixture effects^15–17^, sparse evidence has been reported for the effects of EDC mixtures on the brain^18,19^. Accordingly, in order to appropriately address the mixture issue in chemical risk assessment, more information are needed on the impact of chemical mixtures following exposure protocols mimicking real-life human conditions^20–22^.

By combining epidemiology data from the Swedish Environmental, Longitudinal, Mother and child, Asthma and allergy (SELMA) pregnancy cohort study with advanced biostatistics, Bornehag *et al*^23^. defined a number of EDC mixtures that were associated with adverse outcomes in metabolism and growth, sexual development and neurodevelopment of the prenatally exposed children. In the frame of the EDC-MixRisk Project (https://edcmixrisk.ki.se/), these mixtures were then tested in animal and cellular models by keeping exposure rates comparable to the human situation. The results obtained so far show substantial similarity of the affected health outcomes between humans and experimental models^24–26^. The first neurodevelopment-associated mixture^25^ has been tested in several experimental systems that did not include an *in vivo* mammalian model^24^. This mixture has been expanded with the inclusion of additional potentially-harmful chemicals based on further analysis of the SELMA study data to constitute mixture N1.

In the present study, we describe a refined neurodevelopment-associated mixture, mixture N1, whose components were detected in urine and serum of pregnant women from the SELMA study and were associated with language delay in their 2.5-years old children. The human exposure to mixture N1 was simulated in mice and the long-term effects of mixture exposure were examined in adult offspring at the behavioral and gene expression level. We thus exposed pregnant mice at doses of mixture N1 representing 0×, 0.5×, 10×, 100× and 500× the geometric mean of the levels measured in the SELMA mothers. A battery of behavioral assays was selected to assess anxiety, sociability, learning and stress coping of the adult male and female offspring, as these behaviors comprise a broad phenotype and are frequently targeted by several EDCs^27^. Based on the behavioral outcomes we then chose a number of relevant genes and examined their expression in areas of the limbic system known to be involved in the aforementioned behaviors. From the plethora of potentially implicated genes we favoured genes related to the Hypothalamic Pituitary Adrenal (HPA) axis function since it interferes with the aforementioned behaviors and because we^28–30^ and others^31–33^ have previously shown that it is impacted by early-life exposures to EDCs, such as BPA, a chemical included in mixture N1. Specifically, *Nr3c1* and *Nr3c2* genes encode for corticosterone receptors (GR and MR) two key mediators of HPA axis activity^34,35^ in interaction with CRH and its receptor CRHR1^36–39^. GRs are negatively regulated by FKBP5 that in turn is a common target of EDCs^40–42^. Emotionality and stress coping strategy is also fine-tuned by the serotonin receptors HTR1a and 2a^43^, while the glutamate receptor subunit GRIN2b is implicated in EDC-mediated neurotoxicity and neurodevelopmental disorders in children including language, motor and learning deficits, autism spectrum disorder (ASD), and attention deficit hyperactivity disorder (ADHD)^44,45^. Oxytocin and its receptor are regulators of anxiety-related behaviors and sociability^46–48^, while the expression of many of the abovementioned molecules is controlled by estrogen receptor beta^30,49^. Due to the plasticity of the limbic system to new experiences, the mixture effect on gene expression was examined in matched siblings a) under basal conditions and b) following the behavioral tests, in order to further investigate the potential impact of *in utero* exposure to mixture N1 on the new-experience-induced brain plasticity. Furthermore, both sexes were included in our study in order to detect any sex-specific effects of mixture N1 exposure on behavior and brain gene expression.

## Results

### Mixture N1 composition

By the use of weighted quantile sum (WQS) regression in the SELMA study, we identified eight chemicals of concern associated with language delay in children at the age of 2.5 years, among the 54 compounds/metabolites detected in the urine/serum of their mothers at median pregnancy week 10. The mixture (mixture N1) includes four phthalate diesters (DEP, DBP, BBzP, DIDP/DPHP), three pesticides (pp-DDE, TCP, 3-PBA) and Bisphenol A (Table 1).

**Table 1:** Mixture N1 composition. Geometric means (GM) in pregnant women in SELMA for chemical compounds in the urine or serum and their mixing percentages. DEP: Di-ethyl phthalate, DBP: Di-butyl phthalate, BBzP: Benzyl butyl phthalate, DIDP: Diisodecylphthalate, DPHP: Di(2-propylheptyl) phthalate, Bis(2-propylheptyl) benzene-1,2-dicarboxylate and di(propylheptyl) orthophthalate, BPA: Bisphenol A, TCP: Trichloropyridinol, 3-PBA: 3-Phenoxybenzoic acid, p,p’-DDE: 72-55-9; Dichloro diphenyl dichloro ethylene.

Compound	GM (nmol/mL)	Mixing percentages (% of urine + serum)
DEP	0.03204	44.80
DBP	0.02855	39.92
BBzP	0.00568	7.94
DIDP/DPHP	0.00352	4.92
BPA	0.00047	0.66
TCP	0.00056	0.78
3-PBA	0.00011	0.15
p,p’-DDE	0.00059	0.82
Total	0.0715	100

### *In utero* exposure to mixture N1 modified the behavior of adult offspring

**Figure 1:**
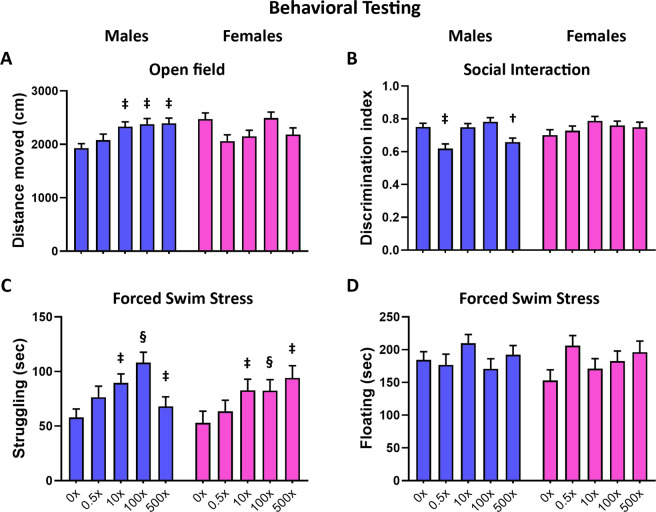
Behavioral responses of adult male (blue bars) and female (magenta bars) mice *in utero* exposed to 0.5×, 10×, 100× and 500× SELMA mothers’ levels of mixture N1 or the vehicle (0×). (**A**) Distance moved in the open field, (**B**) Discrimination index in the social interaction, (**C**, **D**) struggling and floating duration, respectively, during the forced swim stress. Bars represent the estimated marginal means ± SEM. Significance was accepted for P < 0.05. ^†^0.01 < P < 0.05, ^‡^0.001 < P < 0.01, ^§^P < 0.001. Detailed statistics are provided in Supplemental Table S1.

Prenatal exposure to mixture N1 significantly modified the behavioral responses of adult offspring, in a sexually dimorphic way as Mixture-exposed males, but not females, exhibited increased mobility in the open field test (Fig. 1A; sex × treatment interaction: W_4,117_ = 14.627, p = 0.003; for males treatment effect W_4,57_ = 20.485, P < 0.001, post-hoc tests vs. DMSO for groups 10× p = 0.010, 100× p = 0.008 and 500× p = 0.003). Mixture-exposed males exhibited also reduced social interaction with a same sex con-specific (Fig. 1B; sex × treatment interaction: W_4,112_ = 15.351, p = 0.004; for males treatment effect W_4,54_ = 28.576, P < 0.001, post-hoc tests vs. DMSO for groups 0.5× p = 0.002 and 500× p = 0.031). During forced swimming stress (Fig. 1C,D), the exposed offspring of both sexes showed increased struggling towards the walls, indicative of a more active stress coping style (Fig. 1C; treatment effect: W_4,117_ = 21.321, P < 0.001; post-hoc tests vs. DMSO for groups 10× p = 0.009, 100× p = 0.001, 500× p = 0.003). No effects of mixture N1 were observed in the elevated plus maze scores of anxiety or in the novel object location ability in either sex (Supplemental Fig.S1, Table S1).

### In utero exposure to mixture N1 modified the expression of behavior-relevant genes

Based on the behavioral outcomes, we investigated whether the *in utero* treatment modified the expression levels of genes related to these behaviors in the brain of adult offspring, compared to DMSO-treated mice. The effect of mixture N1 on gene expression was analyzed in adult siblings that were either not subjected to any behavioral testing (referred to as Basal animals) or exposed to behavioral testing and sacrificed 30 min from the swimming stress onset (further on referred as Beh. tested animals) vs. the respective DMSO offspring (Basal or Beh. tested). The inclusion of these two conditions (Basal vs. Behavioral testing) aimed to test whether a later life experience (the behavioral examination in adulthood) impacts on gene expression differently upon developmental exposure to mixture N1.

**Figure 2:**
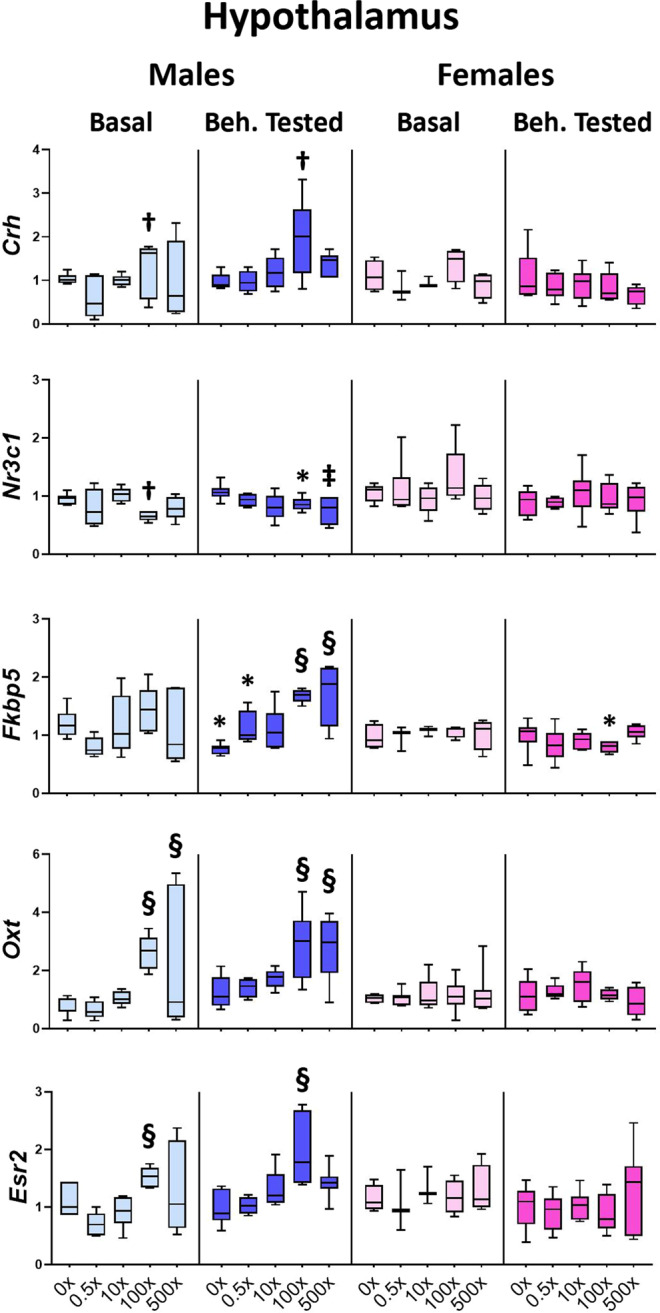
Effect of mixture N1 on hypothalamic gene expression of adult male (blue bars) and female (magenta bars) mice *in utero* exposed to 0.5×, 10×, 100× and 500× SELMA mothers’ levels of mixture N1 or the vehicle (0×). Light colored bars show gene expression under basal conditions (Basal) and dark colored bars the gene expression of matched siblings that underwent the behavioral tests (Beh. Tested). Expression levels were evaluated by qRT-PCR and normalized to *b-actin*. Box plots encompassing values from the 25th to 75th percentile of the data. The horizontal line in the box shows the median value, whereas the horizontal lines above and below the box show the maximal and minimal values, respectively. Three-Way ANOVA with Dunnett’s post hoc test. Significance was accepted for P < 0.05. Significance is shown for the effect of mixture vs. the respective (Basal or Beh. Tested) control. ^†^0.01 < P < 0.05, ^‡^0.001 < P < 0.01, ^§^P < 0.001 as well as for the effect of Beh. Testing vs. the respective Basal group *P < 0.05. Detailed statistics are provided in Supplemental Tables S2 & S2.1.

In the hypothalamus, the expression of all genes studied (*Nr3c1, Crh, Fkbp5, Oxt, Esr2*) was modified by mixture N1 mostly in male offspring (Fig. 2). Reduced levels of *Nr3c1* mRNA were detected in Basal males (treatment effect: F_4,26_ = 3.248, P = 0.031) exposed to 100× mixture (p = 0.040) and in Beh. tested males (F_4,29_ = 3.125, P = 0.010) exposed to 500× mixture (post hoc p = 0.033). In contrast, under both basal and behavioral testing conditions, increased mRNA levels were detected for *Crh* (F_4,58_ = 4.645, P = 0.003) in 100× group (p = 0.011), *Oxt* (F_4,58_ = 9.720, P < 0.001) in 100×(p < 0.001) and 500×(p = 0.001) groups and for *Esr2* (F_4,58_ = 9.066, P < 0.001) in 100× group (p < 0.001). Following the behavioral experience *Fkbp5* levels increased compared to the respective DMSO group (F_4,29_ = 10.774, P < 0.001) in the 100×(p < 0.001) and 500×(p < 0.001) mice. In the DMSO males *Fkbp5* was reduced in Beh. tested animals compared to the Basal DMSO ones (treatment x sex x behavioral testing interaction: F_4,109_ = 2.948, P = 0.024, post hoc p = 0.001), while this was not detected in the mixture-treated offspring. In females, the behavioral experience decreased *Fkbp5* levels (F_4,109_ = 2.948, P = 0.024) in 100× females compared to the respective basal group (p = 0.003). Detailed statistics in Supplementary Tables S2 and S2.1

**Figure 3:**
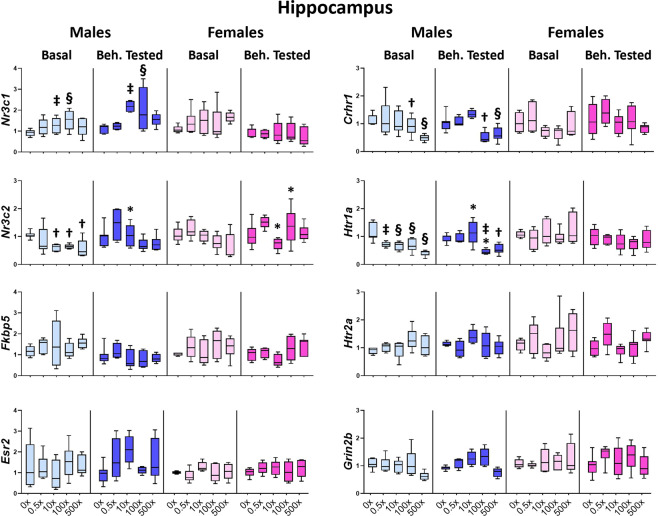
Effect of mixture N1 on hippocampal gene expression of adult male (blue bars) and female (magenta bars) mice *in utero* exposed to 0.5×, 10×, 100× and 500× SELMA mothers’ levels of mixture N1 or the vehicle (0×). Light colored bars show gene expression under basal conditions (Basal) and darkly colored bars the gene expression of matched siblings that underwent the behavioral tests (Beh. Tested). Expression levels of indicated genes were evaluated by qRT-PCR and normalized to *b-actin*. Box plots encompassing values from the 25th to 75th percentile of the data. The horizontal line in the box shows the median value, whereas the horizontal lines above and below the box show the maximal and minimal values, respectively. Three-Way ANOVA with Dunnett’s post hoc test. Significance was accepted for P < 0.05. Significance is shown for the effect of mixture vs. the respective (Basal or Beh. Tested) control. ^†^0.01 < P < 0.05, ^‡^0.001 < P < 0.01, ^§^P < 0.001 as well as for the effect of Beh. Testing vs. the respective Basal group *P < 0.05. Detailed statistics are provided in Supplemental tables S3 & S3.1.

In the hippocampus, the expression of *Nr3c1, Nr3c2, Crhr1* and*Htr1a* was significantly modified by the mixture exposure in male but not female offspring (Fig. 3). Expression of the GR encoding gene (*Nr3c1*) was significantly increased in the hippocampus of both Basal and Beh. tested males (treatment effect: F_4,59_ = 5.563, P = 0.001) exposed *in utero* to 10×(p = 0.002) or 100×(p = 0.001) of mixture N1, as compared to the respective DMSO groups. In contrast, the expression of the MR encoding gene (*Nr3c2*) was decreased (F_4,30_ = 3.771, P = 0.015) in exposed Basal males of the 10×(p = 0.021), 100×(p = 0.038) and 500×(p = 0.012) groups, but this overall reduction in Beh. tested offspring (F_4,29_ = 3.246, P = 0.028) did not reach significance in post hoc comparisons of Beh. tested offspring. An overall effect of mixture N1 was also detected in the ratio of *Nr3c1/ Nr3c2* (F_4,126_ = 5.424. P = 0.001) that was significantly increased in the 10×, 100× and 500× offspring of both sexes (p = 0.034, 0.006 and 0.002, respectively) (Supplementary Fig.S2A and Table S3). The expression of *Crhr1* was reduced in the hippocampus of both Basal and Beh. tested males (F_4,60_ = 9.125, P < 0.001) of the 100×(p = 0.026) and 500×(p = 0.001) groups. Decreased expression of serotonin receptor *Htr1a* was also detected in both Basal (F_4,30_ = 11.628, P < 0.001) for all treated groups, (p = 0.002, 0.001, 0.001, <0.001 for 0.5×, 10×, 100× and 500×, respectively) and Beh. tested males (F_4,29_ = 9.147, P < 0.001) of the 100×(p = 0.006) and 500×(p = 0.024) groups vs. the respective DMSO groups. Accordingly, the ratio of *Htr1a*/*Htr2a* was significantly decreased (F_4,29_ = 7.361, P < 0.001) in 0.5×(p = 0.013), 100×(p = 0.001) and 500×(p < 0.001) Basal male offspring (Supplementary Fig.S2A, Table S3). This difference did not reach significance in post hoc comparisons of Beh. tested offspring, as the *Htr1a*/*Htr2a* ratio in the DMSO group was reduced compared to the Basal DMSO one (treatment x sex x behavioral testing interaction: F_4,109_ = 2.517, P = 0.046, post hoc p = 0.019) (Supplementary Fig. S2A, Table S3.1). In addition, *Nr3c2* levels were modified following behavioral testing in a sex and dose-dependent way (treatment x sex x behavioral testing interaction: F_4,125_ = 2.998, P = 0.022): they were increased in 10× males (post hoc p = 0.025), decreased in 10× females (post hoc p = 0.049) and increased in 100× females (post hoc p = 0.038) compared to their respective Basal groups (Fig. 3, Table S3.1). Moreover, *Htr1a* expression was modified following behavioral testing (F_4,125_ = 3.295, P = 0.014): it was increased in 10× males (p = 0.042), while it was decreased in 100× males (p = 0.049) compared to the respective Basal males (Fig. 3, Table S3.1). No significant effects of the mixture exposure and/or the behavioral experience were detected on the expression of *Fkbp5, Esr2, Htr2a, Grin2b*, in either sex (Fig. 3, Supplementary Tables S3, S3.1).

**Figure 4:**
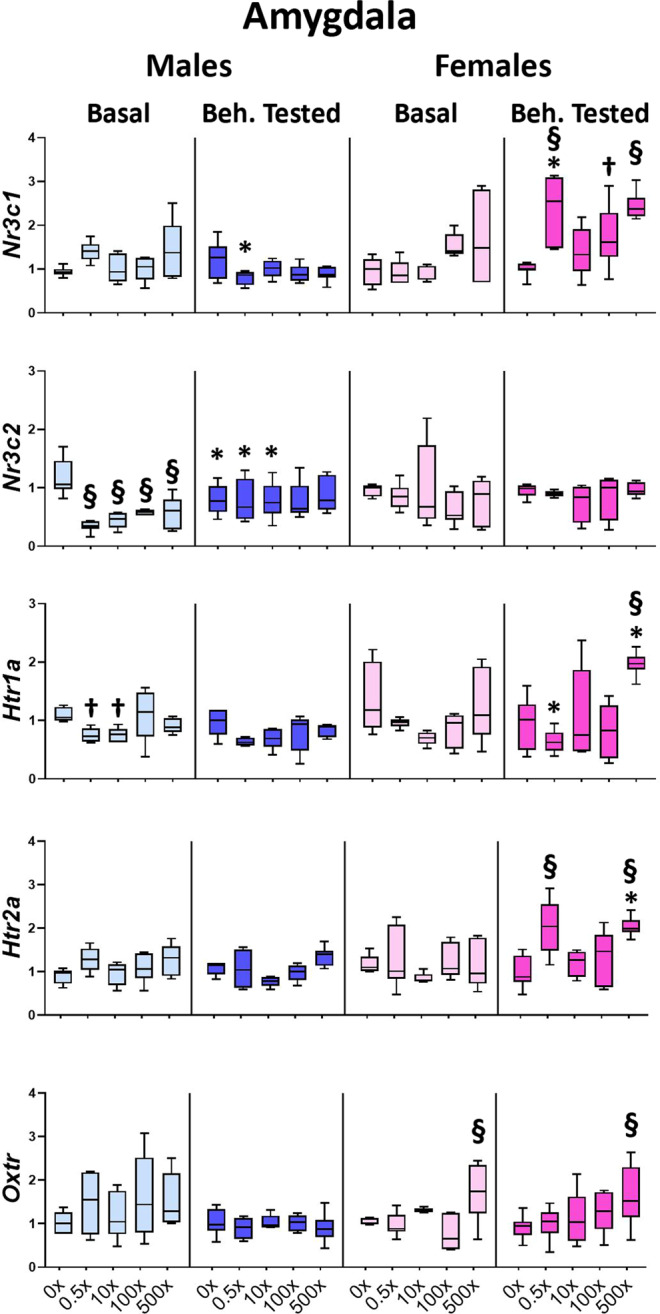
Effect of mixture N1 on amygdalar gene expression of adult male (blue bars) and female (magenta bars) mice *in utero* exposed to 0.5×, 10×, 100× and 500× SELMA mothers’ levels of mixture N1 or the vehicle (0×). Light colored bars show gene expression under basal conditions (Basal) and darkly colored bars the gene expression of matched siblings that underwent the behavioral tests (Beh. Tested). Expression levels of indicated genes were evaluated by qRT-PCR and normalized to *b-actin*. Box plots encompassing values from the 25th to 75th percentile of the data. The horizontal line in the box shows the median value, whereas the horizontal lines above and below the box show the maximal and minimal values, respectively. Three-Way ANOVA with Dunnett’s post hoc test. Significance was accepted for P < 0.05. Significance is shown for the effect of mixture vs. the respective (Basal or Beh. Tested) control.^†^0.01 < P < 0.05, ^§^P < 0.001 as well as for the effect of Beh. Testing vs. the respective Basal group *P < 0.05. Detailed statistics are provided in Supplemental Tables S4 & S4.1.

Amygdala is a limbic area reciprocally communicating with the HPA axis, the hippocampus and the prefrontal cortex in the processing of emotional experiences. *In utero* exposure to mixture N1 either under basal conditions or following behavioral testing significantly modified the expression of all genes examined, in a sexually dimorphic manner. The expression of *Nr3c2* was reduced (F_4,29_ = 17.575, P < 0.001) in all N1-treated groups of Basal males vs. DMSO (p < 0.001 for all groups), whereas the levels of *Nr3c1* were increased in Beh. tested females (F_4,31_ = 10.933, P < 0.001) of the 0.5×(p < 0.001), 100×(p = 0.049) and 500×(p < 0.001) groups vs. Beh. tested DMSO (Fig. 4, Supplementary Table S4). Accordingly, mixture exposure led to increased *Nr3c1*/*Nr3c2* ratio in Basal males (F_4,29_ = 5.404, P = 0.003) of the 0.5×(p = 0.001) and 500×(p = 0.024) groups, as well as in Basal females (F_4,29_ = 7.600, P < 0.001) of the 100×(p = 0.001) and 500×(p = 0.016) groups and in Beh. tested females (F_4,28_ = 4.612, P = 0.007) of the 0.5×(p = 0.014), 100×(p = 0.007) and 500×(p = 0.015) groups, compared to their respective DMSO groups (Supplementary Fig. S2B, Table S4). Mixture exposure increased the expression of *Oxtr* in the amygdala of the 500× females of both Basal and Beh. tested animals (F_4,60_ = 6.279, P < 0.001) compared to the respective DMSO groups (p < 0.001 for both). Serotonin receptor *Htr1a* expression was significantly reduced in mixture-exposed Basal male offspring (F_4,30_ = 3.503, P = 0.020) of the 0.5×(p = 0.037) and 10×(p = 0.043) groups, but this reduction did not reach significance in Beh. tested males. In contrast, in Beh. tested females increased levels of *Htr1a* (F_4,30_ = 8.933, P < 0.001) in the 500× group (p = 0.001) and *Htr2a* (F_4,30_ = 7.895, P < 0.001) in the 0.5× and 500× groups (p = 0.001 for both) were detected vs. the respective DMSO offspring (Fig. 4, Supplementary Table S4). The modified expression levels of *Htr1a* and *Htr2a* resulted in an overall reduction in the *Htr1a*/*Htr2a* ratio (F_4,120_ = 5.927, P = 0.017) in the 0.5×(p = 0.002) groups of both Basal or Beh. tested males and females (Supplementary Fig. S2B, Supplementary Table S4).

In addition, in the amygdala the behavioral testing differentially affected the gene expression in a dose and sex-specific way as follows: *Nr3c1* levels were reduced in 0.5× Beh. tested males (treatment x sex x behavioral testing interaction: F_4,122_ = 6.840, P < 0.001, post hoc p = 0.002) while they were increased in 0.5× Beh. tested females (post hoc p = 0.005), compared to the respective Basal groups (Fig. 4, Supplementary Table S4.1). *Nr3c2* levels were differentially expressed between the Beh. tested and Basal groups (treatment x sex x behavioral testing interaction: F_4,118_ = 2.501, P = 0.047): in males they were reduced in the Beh. tested *vs*. Basal DMSO offspring (p = 0.026), whereas they were increased in the corresponding groups of 0.5×(p = 0.027) and 10×(p = 0.036) (Fig. 4, Supplementary Table S4.1). Consequently, the *Nr3c1*/*Nr3c2* ratio following behavioral testing was modified (treatment x sex x behavioral testing interaction: F_4,118_ = 6.063, P < 0.001), compared to the respective ratio of Basal offspring: in males it was increased in DMSO (p = 0.004) and decreased in 0.5×(p = 0.029) offspring, whereas in females the ratio was increased in 0.5×(p = 0.008) mice (Fig.S2B, Supplementary Table S4.1). Additionally, in Beh. tested females, the *Htr1a* expression was reduced in the 0.5× group (treatment x sex x behavioral testing interaction: F_4,121_ = 2.976, P = 0.023, post hoc p = 0.009), while both *Htr1a* (F_4,121_ = 2.976, P = 0.023, post hoc p = 0.016) and *Htr2a* expression (F_4, 120_ = 3.407, P = 0.012, post hoc p = 0.002) was increased in 500× group, compared to the respective Basal groups (Fig. 4, Supplementary Table S4.1).

**Figure 5:**
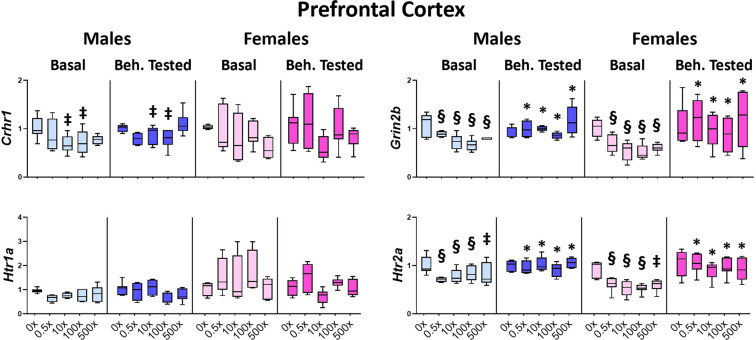
Effect of mixture N1 on prefrontal cortex gene expression of adult male (blue bars) and female (magenta bars) mice *in utero* exposed to 0.5×, 10×, 100× and 500× SELMA mothers’ levels of mixture N1 or the vehicle (0×). Light colored bars show gene expression under basal conditions (Basal) and darkly colored bars the gene expression of matched siblings that underwent the behavioral tests (Beh. Tested). Expression levels of indicated genes were evaluated by qRT-PCR and normalized to *b-actin*. Box plots encompassing values from the 25th to 75th percentile of the data. The horizontal line in the box shows the median value, whereas the horizontal lines above and below the box show the maximal and minimal values, respectively. Three-Way ANOVA with Dunnett’s post hoc test. Significance was accepted for P < 0.05. Significance is shown for the effect of mixture vs. the respective (Basal or Beh. Tested) control. ^‡^0.001 < P < 0.01, ^§^P < 0.001 as well as for the effect of Beh. Testing vs. the respective Basal group *P < 0.05. Detailed statistics are provided in Supplemental Tables S5 & S5.1.

In the prefrontal cortex, the levels of *Crhr1* were significantly decreased in both Basal and Beh. tested males (treatment effect: F_4,56_ = 3.585, P = 0.012) of the 10×(p = 0.023) and 100×(p = 0.011) groups, compared to the respective DMSO groups, but not in female offspring (Fig. 5, Supplementary Table S5). In both sexes, the expression levels of serotonin receptor *Htr2a* were significantly reduced in Basal animals (F_4,57_ = 6.550, P < 0.001) from all mixture-treated groups, vs. the respective Basal DMSO animals (p < 0.001 for the groups 0.5×−100× and p = 0.003 for the 500×). The same reduction was detected in all mixture-exposed groups of Basal animals for the glutamate receptor subunit *Grin2b* (F_4,57_ = 14.384, P < 0.001, post hoc p < 0.001 for all groups). However, these reductions in *Htr2a* and *Grin2b* were not detected in the Beh. tested siblings of the aforementioned Basal animals due to behavioral experience-induced changes. Specifically, *Htr2a* expression was increased in Beh. tested animals of all N1-exposed groups (treatment x behavioral testing interaction: F_4,119_ = 2.583, P = 0.042, post hocs p < 0.001, 0.001, 0.001 and 0.002, respectively). Similarly, *Grin2b* levels were increased in Beh. tested offspring of vs. their respective Basal ones (treatment x behavioral testing interaction: F_4,119_ = 2.961, P = 0.023, post hocs p = 0.008, 0.002, 0.001 and 0.003, respectively) (Fig. 5, Table S5.1). On the contrary, Beh. tested DMSO-treated animals did not differ from the respective Basal ones. The expression levels of serotonin receptor *Htr1a* did not differ significantly.

### Effects on stress hormone levels, adrenal weight and gene expression

Due to the inclusion of the forced swimming stress in the behavioral tests, we examined potential effects of *in utero* exposure to mixture N1 on stress hormone levels and mediators of steroidogenesis and secretion. Three-way ANOVA for the effects of mixture N1, sex and behavioral testing showed no significant effects of mixture exposure on serum corticosterone or ACTH levels, measured under basal conditions and at 30 min from the stress onset (Table 2). On the other hand, *in utero* exposure to mixture N1 had an overall effect on adrenal weight (F_4,117_ = 4.407, P = 0.003) and adrenal weight /body weight ratio (adrenal index,F_4,117_ = 5.015, P = 0.001) that was significantly increased in the adult offspring (10×, 100×, 500×) of both sexes (Table 2). No effect of mixture was detected on body weights.

**Table 2:** Serum ACTH (pg/ml) and Corticosterone levels (ng/ml) as well as adrenal weight (mg), body weight (gr) and adrenal index (adrenal weight/body weight) of adult male and female mice exposed *in utero* to Mixture N1 at 0×, 0.5×, 10×, 100× and 500× SELMA mothers’ levels. Parameters were determined in mice sacrificed under BASAL conditions and at 30 minutes from the onset of stress (BEH. TEST). Values represent Means +/− SEM. The number of samples from different litters is given in parentheses. Three-way ANOVA, Dunnett’s post hoc; *P < 0.05, **P < 0.01, ***P < 0.001.

ACTH		0 ×	0.5×	10×	100×	500×
MALES	BASAL	62.07 +/− 6.9 (7)	71.05 +/− 7.4 (4)	54.98 +/− 8.7 (6)	55.4 +/− 7.8 (6)	62.15 +/− 5.6 (6)
	BEH.TEST	107.5 +/− 22.6 (6)	153.9 +/− 56.1 (4)	129.2 +/− 28.3 (6)	125.8 +/− 42.4 (6)	133.9 +/− 33.2 (6)
FEMALES	BASAL	50.4 +/− 11.3 (5)	47.7 +/− 4.4 (7)	56.7 +/− 7.8 (5)	44.3 +/− 2.4 (7)	39.6 +/− 1.6 (6)
	BEH.TEST	91.9 +/− 18.3 (6)	94.5 +/− 17.9 (6)	139.5 +/− 57.8 (6)	54.4 +/− 11.7 (6)	56.9 +/− 8.9 (6)
**Corticosterone**						
MALES	BASAL	26.3 +/− 10.8 (7)	70.9 +/− 5.6 (4)	50.9 +/− 11.9 (6)	36.0 +/− 9.3 (6)	45.9 +/− 16.7 (6)
	BEH.TEST	608.9 +/− 32.6 (7)	616.3 +/− 33.8 (4)	497.9 +/− 41.9 (6)	701.9 +/− 107.9 (6)	539.4 +/− 40.7 (7)
FEMALES	BASAL	67.7 +/− 23.0 (6)	99.9 +/− 41.1 (6)	100.3 +/− 29.9 (6)	84.7 +/− 28 (6)	139.7 +/− 22.2 (6)
	BEH.TEST	1392.7 +/− 118.4 (6)	1029.3 +/− 120 (6)	1229.4 +/− 139.5 (6)	1642.7 +/− 62.2 (6)	1652.5 +/− 35.2 (6)
**Adrenal weight (mg)**						
MALES	BASAL	13.29 +/− 0.94 (7)	19.69 +/− 2.83 (4)	20.84 +/− 3.05** (5)	16.91 +/− 2.63* (6)	20.11 +/− 3.34* (6)
	BEH.TEST	12.79 +/− 0.45 (7)	14.92 +/− 2.32 (4)	16.52 +/− 1.46** (6)	15.24 +/− 1.29* (6)	14.39 +/− 1.5* (7)
FEMALES	BASAL	14.44 +/− 1.68 (6)	13.76 +/− 0.8 (6)	19.44 +/− 2.48** (6)	20.51 +/− 3.65* (6)	21.29 +/− 2.42* (6)
	BEH.TEST	12.47 +/− 0.39 (6)	15.33 +/− 1.46 (6)	17.79 +/− 1.58** (6)	14.19 +/− 1.49* (6)	14.28 +/− 1.5* (6)
**Body weight (gr)**						
MALES	BASAL	24.81 +/− 0.49 (7)	27.52 +/− 0.38 (4)	25.74 +/− 0.58 (5)	25.56 +/− 0.96 (6)	25.25 +/− 1.29 (6)
	BEH.TEST	24.12 +/− 0.41 (7)	24.73 +/− 0.49 (4)	25.18 +/− 0.67 (6)	24.55 +/− 0.99 (6)	24.33 +/− 0.92 (7)
FEMALES	BASAL	19.16 +/− 0.42 (6)	19.07 +/− 0.27 (6)	19.67 +/− 0.19 (6)	19.75 +/− 0.33 (6)	19.46 +/− 0.58 (6)
	BEH.TEST	18.73 +/− 0.27 (6)	19.4 +/− 0.37 (6)	19.56 +/− 0.21 (6)	19.08 +/− 0.2 (6)	18.93 +/− 0.59 (6)
**Adrenal index**						
MALES	BASAL	0.54 +/− 0.03 (7)	0.72 +/− 0.1 (4)	0.8 +/− 0.1*** (5)	0.66 +/− 0.1* (6)	0.78 +/− 0.11** (6)
	BEH.TEST	0.53 +/− 0.02 (7)	0.61 +/− 0.09 (4)	0.69 +/− 0.03*** (6)	0.62 +/− 0.05* (6)	0.59 +/− 0.06** (7)
FEMALES	BASAL	0.69 +/− 0.03 (6)	0.72 +/− 0.05 (6)	0.99 +/− 0.13*** (6)	1.04 +/− 0.19* (6)	1.09 +/− 0.11** (6)
	BEH.TEST	0.67 +/− 0.02 (6)	0.8 +/− 0.09 (6)	0.91 +/− 0.08*** (6)	0.69 +/− 0.04* (6)	0.7 +/− 0.03** (6)

**Figure 6:**
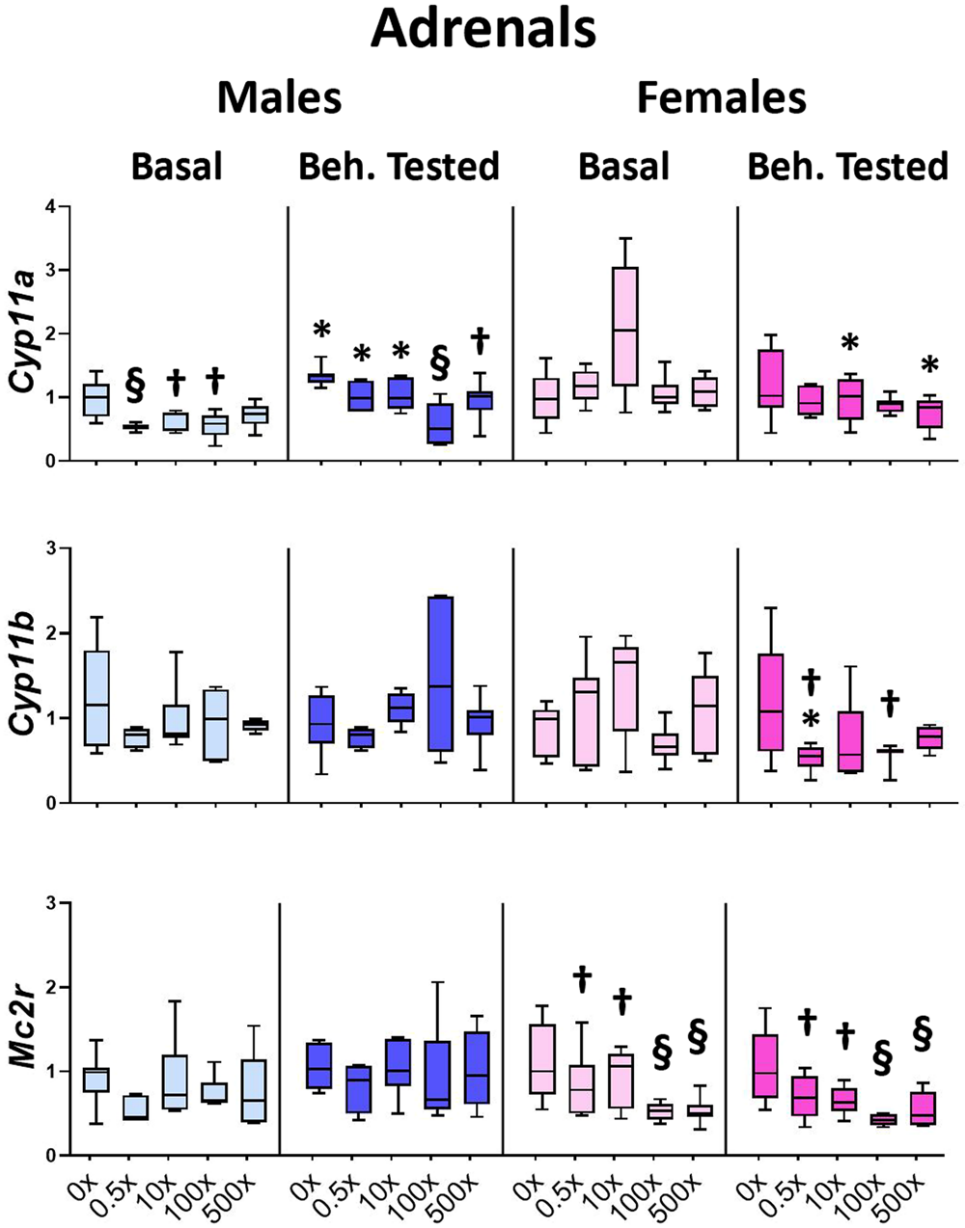
Effect of mixture N1 on adrenal gene expression of adult male (blue bars) and female (magenta bars) mice *in utero* exposed to 0.5×, 10×, 100× and 500× SELMA mothers’ levels of mixture N1 or the vehicle (0×). Light colored bars show gene expression under basal conditions (Basal) and darkly colored bars the gene expression of matched siblings that underwent the behavioral tests (Beh. Tested). Expression levels of indicated genes were evaluated by qRT-PCR and normalized to *b-actin*. Box plots encompassing values from the 25th to 75th percentile of the data. The horizontal line in the box shows the median value, whereas the horizontal lines above and below the box show the maximal and minimal values, respectively. Three-Way ANOVA with Dunnett’s post hoc test. Significance was accepted for P < 0.05. Significance is shown for the effect of mixture vs. the respective (Basal or Beh. Tested) control. ^†^0.01 < P < 0.05, ^§^P < 0.001 as well as for the effect of Beh. Testing vs. the respective Basal group *P < 0.05. Detailed statistics are provided in Supplemental Tables S6 & S6.1.

In the adrenal glands (Fig. 6, Supplementary Table S6), the expression of the rate-limiting steroidogenic enzyme *Cyp11a1* was significantly reduced in Basal (treatment effect: F_4,29_ = 5.559, P = 0.002) males of the 0.5×−100×(p = 0.001, 0.023, 0.030, respectively), as well as in Beh. tested (F_4,29_ = 6.921, P = 0.001) males of the 100×(p < 0.001) and 500×(p = 0.029) groups. No effect of mixture on *Cyp11a1* levels was detected in the female groups with the exception of the 10× sub-group of females at the low estrogen phases of the menstrual cycle (F_4,29_ = 5.005, P = 0.004, post hoc p = 0.019). However, following behavioral testing *Cyp11a1* levels were reduced in 10× and 500× females compared to the respective Basal groups (treatment x sex x behavioral testing interaction: F_4,123_ = 3.765, P = 0.007, post hocs p = 0.032, p = 0.029, respectively) (Fig. 6 and Supplementary Table S6.1). Additionally, while the levels of *Cyp11a1* were increased (vs. Basal) in Beh. tested males (F_4,123_ = 3.765, P = 0.007) of the DMSO (p = 0.012), 0.5×(p = 0.037 and 10×(p = 0.013) groups, they failed to do so in the groups of 100× and 500× Beh. tested offspring (Fig. 6, Supplementary Table S6.1). The expression of *Cyp11b1*, which encodes for the critical enzyme of corticosterone synthesis, was significantly reduced (treatment effect: F_4,33_ = 3.268, P = 0.029) in the groups of 0.5×(p = 0.016) and 100×(p = 0.019) of Beh. tested females exposed to mixture N1 vs. Beh. tested DMSO animals; for the 0.5× females, this is due to a reduction in expression levels following behavioral testing, compared to the levels of 0.5× Basal females (treatment x sex x behavioral testing interaction: F_4,124_ = 3.262. P = 0.015, post hoc p = 0.026) (Fig. 6, Supplementary Table S6.1). The levels of *Mc2r* mRNA, encoding for the ACTH receptor in adrenals, were also significantly reduced in all mixture-exposed female groups, irrespective of behavioral testing (treatment effect: F_4,63_ = 9.035, P < 0.001, pot hocs for 0.5× p = 0.048, 10× p = 0.040, 100×<0.001 and 500×<0.001).

No significant effects of mixture or behavioral testing were detected on the expression of *Pomc* or *Crhr1* in the pituitary (Supplementary Fig.S3).

### Principal component analysis

**Figure 7:**
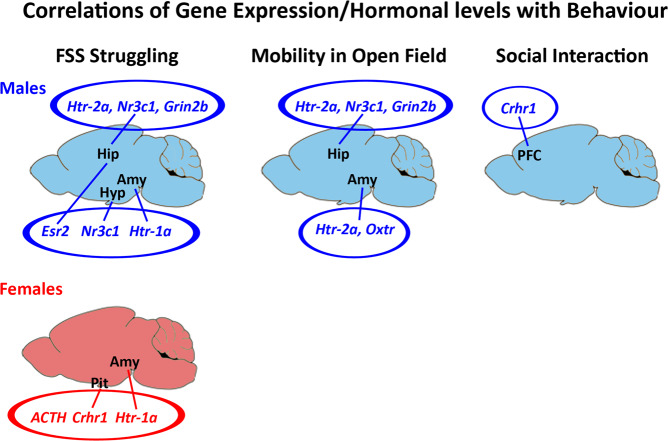
Graphical presentation of the Principal Component Analysis derived clusters of correlated gene/hormonal levels with the affected behaviors in adult offspring *in utero* exposed to mixture N1. Amy: Amygdala; Hip: Hippocampus, Hyp: Hypothalamus, Pit: Pituitary, PFC: Prefrontal cortex.

In males, principal component analyses indicated that the levels of struggling behavior during the forced swim stress clustered with two sets of genes, indicative of co-variation: One cluster included struggling behavior (coefficient value (CV) = 0.491) and the hippocampal levels of *Nr3c1* (CV = 0.858), *Htr2a* (CV = 0.87) and *Grin2b* (CV = 0.659), while the other included struggling behavior (CV = −0.47) and the hypothalamic levels of *Nr3c1* (CV = 0.910), the amygdalar levels of *Htr1a* (CV = 0.421) and the hippocampal levels of *Esr2* (CV = −0.464) (Fig. 7). Moreover, PCA revealed that mobility of males in the open field clustered also with two sets of genes: One cluster included mobility (CV = 0.606) and the amygdalar levels of *Oxtr* (CV = −0.852) and *Htr2a* (CV = 0.628) and the other included mobility (CV = 0.446) and the hippocampal levels of *Htr2a* (CV = 0.880), *Nr3c1* (CV = 0.866) and *Grin2b* (CV = 0.478). In addition, PCA showed that the levels of social interaction of males co-varied with the prefrontal levels of *Crhr1* (CV = −0.669 and CV = 0.936, respectively). Interestingly, for females, PCA identified only a clustering of their levels of struggling behavior during FSS (CV = −0.564) with the levels of ACTH (CV = 0.751), the levels of *Crhr1* in the pituitary (CV = 0.456) and the levels of *Htr1a* in the amygdala (CV = −0.454). The PCA plots of the complete set of genes analyzed can be found in Supplementary Fig.S4.

## Discussion

Previous epidemiological and experimental evidence have associated perinatal exposure to individual EDCs, such as BPA and phthalates, with neurodevelopmental modifications that may impact later life brain function and behavior^3,4,7–9^. However a limited number of experimental studies have so far examined the impact of mixtures of EDCs on the brain^18,19^. Another gap in the current literature is the absence of experimental studies simulating the conditions of human gestational exposure to mixtures of EDCs that have been previously associated with a particular neurodevelopmental dysfunction in children. To address this issue, we have used a mixture of EDCs or suspected EDCs that are associated with adverse neurodevelopmental outcomes in children (language delay)^25^ to study its long term neurobehavioral effects following gestational exposure in mice. Although the language delay cannot be modeled in animals, *in utero* exposure of mice to mixture N1 significantly modified their behavior and brain transcriptional activity in adulthood. The mixture-induced changes, detected at 0.5×− 500× of the SELMA pregnant women exposure levels, appear sexually dimorphic, brain area specific and some of them are modified by adult life experiences.

The male offspring phenotype is reminiscent of hyperactivity as deduced from the increased struggling during forced swimming stress and the increased locomotion in the open field. Hyperactivity has also been reported in children^9^ and rodents exposed to bisphenol A^12^, as well as in rat pups exposed to a mixture of flame retardants and phthalates^50^. Notably, hyperactivity is a trait often seen in animal models of neurodevelopmental disorders including ADHD, schizophrenia and ASD^51^. In addition, reduced social interaction was observed in male offspring exposed to the N1 mixture. This phenotype could be attributed either to reduced interest for other con-specifics or to social phobia and withdrawal. Aberrant social behavior has been also reported in rats chronically exposed to phthalates^52^ and the emergence of ASD in humans has been linked to early life exposure to phthalates or other anthropogenic environmental toxicants^53^.

Gene expression changes measured in the male offspring exposed to mixture N1 indicate a hyperactive stress response system and are consistent with their behavioral phenotype. Specifically, the increased *Crh* and the reduced *Nr3c1* levels in the hypothalamus suggest a heightened stimulation of the HPA axis and a reduced negative feedback, respectively^35^. *Crh* overexpression in mice leads to increased active coping behavior in the forced swimming paradigm^37^, whereas hypothalamic *Crh* depletion has anxiolytic effects in the open field test^38^. The offspring hyperactivity is further primed following the behavioral testing experience as indicated by the higher levels of hypothalamic *Fkbp5*, encoding a negative regulator of GR activation^41,42^. The increased HPA axis activity detected in male offspring may also lead to social phobia and reduced social interaction of these animals. The increased oxytocin expression detected in the hypothalamus of mixture-exposed males does not necessarily connect with the social interaction deficit of these offspring. The enhancing role of oxytocin in social behavior has been documented particularly regarding hippocampal circuits for memory retention^54^ and this role is facilitated by estrogen receptors^55^. However, other hormones, such as vasopressin, and neurotransmitters at different brain areas seem to have a role in sociability of rodents. In support of this, oxytocin-KO male mice exhibit altered hippocampal gene expression and a higher social interaction score than their wild type counterparts^56^. Considering the heightened HPA stimulation of the mixture N1male offspring, the increased levels of oxytocin detected in their hypothalamus could represent a compensatory mechanism towards resilience, based on the reciprocal interplay between oxytocin and CRH^57^. Notably, sexually dimorphic effects on the expression of hypothalamic oxytocin and estrogen receptors have been also reported following gestational BPA exposure in rats^58^.

The hippocampus is a limbic area that regulates the stress axis as well as learning and memory processes. The ratio of the two types of corticosterone receptors GR and MR in this area fine-tunes the different phases of the corticolimbic stress response with MRs having a critical contribution in optimizing coping strategy^34^. GR gene expression is prone to various developmental insults including EDCs^28–33^. The detected imbalance of *Nr3c1*/*Nr3c2* expression ratio in male offspring exposed to mixture N1 suggests a disturbed GR/MR control over the limbic‐cortical stress pathways and a compromised stress response. The modified ratio of the serotonin receptors *Htr1a* and *2a* in favor of the *Htr2a* that is associated with the adoption of an active coping style^43^ could further support the exaggerated active behavioral response of these animals. The similarity in the pattern of male gene expression in the hippocampus and amygdala (reduced *Nr3c2* and *Htr1a* expression) suggests a concerted action of these two limbic structures in the observed behavioral phenotypes. On the other hand, gene expression modifications towards resilience were also detected in male offspring, such as the increased levels of *Oxt* in the hypothalamus to compensate the increased *Crh* levels and the reduced levels of *Crhr1* in the hippocampus and the prefrontal cortex and thus lower anxiogenic stimulation^39^.

In contrast to males, female offspring exposed to mixture N1 exhibited no alterations in the expression of the analyzed genes in the hypothalamus or the hippocampus, a finding compatible with the milder behavioral alterations detected in these animals. However, the increased amygdalar *Nr3c1* expression and the increased *Nr3c1/ Nr3c2* ratio in both the hippocampus and amygdala, could explain the detected alterations in the stress coping of these animals. On the other hand, the increased expression of *Oxtr* in the amygdala may have conferred to the alleviation of anxiety-related impact on female locomotion and sociability^47,48^ in which no significant changes were detected vs. DMSO-treated animals. Notably, in female amygdala, the increased expression of *Nr3c1* as well as that of the serotonin receptors *Htr1a* and *2a* were detected only as a result of the behavioral testing, suggesting the presence of long term effects of gestational exposure to mixture N1 that are manifested only following exposure to challenges/stressors in adulthood.

*Grin2b* expression has been associated with neurodevelopmental disorders^44^ and epigenetic modifications on its gene have been detected following BPA exposures^45^. The prefrontal cortex is continuously integrating information processed by the HPA axis and the limbic system to adjust executive functions and to maintain behavioral homeostasis. In this area, mixture N1 exposure decreased the expression of *Htr2a* and *Grin2b* in both sexes under basal conditions. Interestingly, the mixture-induced reduction in the levels of *Htr2a* and *Grin2b* in the prefrontal cortex was not detected in the Beh. tested animals due to an increase in the mixture-treated groups not found in the DMSO group. This observation further implies the presence of long term effects of gestational exposure to mixture N1 that are manifested only following challenges later in life.

The developing adrenal gland is a target tissue for many EDCs, including phthalates and BPA^29,59,60^. In the present study, gestational exposure to mixture N1 impacted adrenal physiology of the offspring in terms of adrenal weight, steroidogenic enzymes and ACTH receptors. Yet, no significant effect of the mixture was detected on serum corticosterone or ACTH levels, both under basal conditions and at 30 min from stress onset. However, a stressor-induced change in stress hormone levels in mixture-exposed offspring cannot be excluded based on the one time-point measurements following stress exposure, and a more detailed investigation is required, which was beyond the scope of the present study. Accumulating evidence suggests that the effects of individual EDCs can differ from the ones they have as part of a mixture^19^. Accordingly, no direct comparisons can be made on the toxicity-relevant doses of individual chemical disruptors and their combination. The fact that our results on adrenal weight are in agreement with those found in developmentally BPA exposed rats^29^ and mice^60^, whereas the ones on steroidogenic enzymes and corticosterone levels are not similar to those reported for BPA exposures^29,60,61^, further support the differential impact of individual chemicals compared to mixtures.

In the present study we examined the behavioral phenotype of mice developmentally exposed to mixture N1 using an array of behavioral tests and we also determined the expression of a handful of genes known to influence these behaviors. Given that behavioral responses are regulated by a plethora of genes expressed in different brain areas, we applied Principal component analysis (PCA) to reveal gene clusters associated with the observed behavioral outcomes. Correlations implicated mostly the receptors for glucocorticoids, corticotropin and serotonin. These molecules are important regulators of the stress response and emotionality, which further supports that the identified correlations are biologically relevant. Yet, we cannot exclude the important role of other genes not examined in this study or a potential impact of EDCs on the maternal behavior of exposed dams, which could affect both behavior and stress related gene expression in their offspring;^62^ factors that obviously worth further survey.

Interestingly, PCA indicated that in the two sexes different sets of genes and hormones were correlated with active coping in FSS. This implies that, although prenatal EDC exposure leads to similar alterations in the FSS behavior in the two sexes (increased struggling behavior), the underlying biochemical/molecular pathways are diverse. This finding corroborates the view that effects of EDC exposure should be assessed in both females and males, as the implicated cellular pathways might be dissimilar. Overall the present study provides evidence that a human health-relevant mixture of EDCs can lead to long term alterations in mice behavior by modifying gene expression in their brains. Given the increasing concern regarding the long term health consequences stemming from early-life exposures to anthropogenic toxicants, the integrated use of epidemiology and experimental biology tools may be helpful to identify the implicated molecular events and improve the risk assessment strategies.

## Methods

SELMA is a pregnancy cohort conducted in Sweden. All research was performed in accordance with relevant guidelines/regulations. All activities in SELMA meet Swedish national law and ethics requirements regarding recruitment of participants (children and families), collection of human samples, health examination, collection of data by the use of interviews, questionnaires, registers, etc., biobanking of human samples and analyses of biosamples, storing of data in a database, and principles for publications and reports. Participation in the SELMA study was strictly voluntary and informed consent was obtained from all the participants involved. The recruitment procedure and consent forms have been approved by the ethics board of Uppsala, Sweden (DNR 2007/062 and DNR 2015/177). SELMA is collecting and storing sensitive data in a database. All data/information and samples in the database is coded (bar-code) according to Swedish law and European GDPR 2016/679.

To establish Mixture N1, we used data for the levels of 54 chemicals with known or suspected EDC action (compounds or metabolites) in urine and serum from 2,354 women in the SELMA study at median pregnancy week 10; 41 of these compounds were identified above level of quantification (LOQ) in at least 50% of the mothers. For the neurodevelopmental tests performed in children at 2.5 years of age, data were assessed from a standardized nurse examination in combination with parentally completed questionnaires (N = 1,113)^8^. By the use of weighted quantile sum (WQS) regression, we identified among the selected 41 chemicals, those associated with language delay in children at 2.5 years of age (chemicals of concern).We then estimated the daily intake (DI) of identified urinary chemicals of concern by the use of data from 2,300 pregnant women, the plasma concentrations from the DI estimates, and finally the geometric means of both urinary and serum of these chemicals to establish the mixing proportions. In the final step we established a real mixture (Mixture N1) to be tested in the animal model.

Phthalates’ active monoesters^63^ were used to prepare the mixture for mice. The chemicals were purchased from the following sources: Bisphenol A (BPA; 99%), Dimethylsulfoxide (DMSO; 99.9%), Monobenzyl phthalate (MBzP; 98%), 3-Phenoxybenzoic acid (3-PBA; 98%), and Trichloropyridinol (TCP; 99%)were obtained from Sigma-Aldrich Inc. (St. Louis, MO, USA). Monoethyl phthalate (MEP; 98%)and mono-iso-decyl phthalate (MiDP; 98%) were obtained from Toronto Research Chemicals (North York, ON, Canada).Mono-(2-propylheptyl)-phthalate(MPHP, 99%) were synthesized by Novandi Chemistry AB, Södertälje, Sweden. Monobutyl phthalate (MBP; 95%) was purchased from TCI, Tokyo Chemical Industry Co., Ltd (Japan). pp’DDE was synthesized in house by Åke Bergman from DDT. For MIX N1, 1 M solutions in DMSO were prepared of each of the chemicals: BPA, MEP, MBP, BBzP, MIDP, MPHP, 3-PBA, and TCP. A 50/50 mixture of MIDP and MPHP was used. Thereafter, the 1 M solutions were mixed in proportions as described in Table 1. The mixtures were then sent out as 1 M stocks.

### Animals and experimental design

Animal handling and all experiments were performed in accordance with relevant guidelines and regulations (European Communities Council Directive of 22 September 2010; 2010/63/EU). All procedures and experiments were approved by the Ethical Licensing Committee of the Prefecture of Attica-Veterinary department (#4783). All efforts were made to minimize the number of animals used and to reduce their suffering.

Two month old C57/BL6 mice breeders were purchased from the Hellenic Pasteur Institute (Athens, Greece) and were acclimatized for two weeks before use under standard animal house conditions. All animals were offered a phytoestrogen-deficient pellet food (Altromin 1324 P, Lage, Germany) and tap water *ad libitum*. The lack of phthalate and BPA contaminants in mice food or water was verified in a pilot study prior as previously^26^.

Pregnant mice were exposed to the vehicle (DMSO) or to the mixture at doses of 0.5×, 10×, 100× and 500× hsc, where times of “human serum concentration” represents the exposure concentrations relative to the geometric mean of the concentrations measured in pregnant women in the SELMA cohort study. Accordingly, pregnant mice were exposed daily throughout pregnancy to 0.001, 0.22, 2.2 or 11 mg/kg bw of mixture N1, respectively. The dosing per day for each component is shown in Supplementary TableS 7.The stock solutions were aliquoted in volumes of 10 μl in 0.2 ml 100% polypropylene Eppendorf tubes and stored at −20 °C until use. Working solutions of different doses were prepared using DMSO 99.9% purity, Sigma-Aldrich Inc.). The daily dose of the mixture was administered to the individually- housed dams upon pipetting it on an organic cornflake as previously described^26^. DMSO intake did not exceed 0.25 μl/gr bw/day.

Pregnant dams were weighted every 3 days to verify pregnancy and to adjust the exposure dosing. Six cohorts of breeding were included in the study, each containing all animal groups. The total number of litters used was 30. The range of litter sizes and the sex composition per treatment group were as follows: DMSO 7-10 pups (25 males, 21 females), 0.5 × 7–12 pups (31 m, 26 f), 10 × 6–10 pups (26 m, 22 f), 100 × 7–10 pups (27 m, 23 f), 500 × 5–11 pups (30 m, 25 f). The sex ratio did not differ among groups F_4,29_ = 0.030, P = 0.998). In order to behaviorally characterize the offspring in adulthood (PND90), one - three animals per sex and litter from each exposure group were randomly selected to undergo behavioral testing and the rest to remain undisturbed (Basal animals). The number of animals assigned for the behavioral tests per treatment group and sex (m, f), were as follows: DMSO 14 m,12 f; 0.5×10 m, 12 f; 10×12 m 12 f; 100×10 m, 13 f; 500 × 12 m, 11 f. All Behaviorally tested animals were subjected to the same battery of behavioral tasks in an order taking into account a. the compatibility of tests (e.g. open field can serve as an accommodation phase for social interaction) and b. the degree of stressfulness of the task i.e. forced swimming stress being the last one employed. Behavioral tests were conducted, over a period of eight days in the following order: elevated plus maze (day 1), open field (day 2), social interaction (days 3 and 4), novel object location (day 5). The tested animals were then left undisturbed for the next two days (days 6 and 7) and then (day 8) were subjected to forced swimming stress (FSS).The animals were sacrificed 30 min from the FSS onset, in order to investigate their hormonal response to an acute stress. Littermates not behaviorally tested (Basal) were sacrificed along with those Behaviorally tested. Euthanasia was conducted between 9.00 to 12.00 am under isoflurane anesthesia. Trunk blood was collected for hormone level determination. The pituitary and the brain were rapidly removed from the scull and the relevant brain areas (hypothalamus, hippocampus, amygdala and prefrontal cortex) were isolated on a frozen surface, snap-frozen in dry ice-cooled isopentane and transferred to −80^0^C until use. The adrenals were placed in pre-weighted vials and immediately frozen. The estrous phase of females was recorded by macroscopic observation during the behavioral tests in order to minimize animal stress and by vaginal smears at sacrifice.

### Behavioral tests

All behavioral tests were performed during the light period between 9.00 to 12.00 am in order to avoid “ceiling”-effects in behaviors such as mobility since mice show increased activity during the dark phase of the diurnal cycle, as well as to be able to compare our results with those in the literature and establish a trustworthy base-line for the DMSO-treated group. Behaviors were recorded using an Everio GZ-MS110 Memory Camera (*JVC*) and the recorded videos were analyzed by three different observers, blind to the treatment of each subject.

### Elevated plus maze

The test was conducted according to Segklia *et al*.^64^. The number of entries and the time spent (sec) in either the open or the enclosed arms was counted. The time spent in the open arms is indicative of the level of anxiety of the mouse.

### Open field test

The test was conducted in a square Plexiglas box (40 × 40 × 40 cm); the mouse was gently placed in the center of the arena and left to move freely for 5 min to capture the critical components of general exploratory locomotion^65^. The total locomotion of the subjects was evaluated by using the Ethovision XT-7 software (NOLDUS). The time spent in the center and in the periphery of the arena, indicating reduced or increased anxiety levels, respectively, were also measured. In addition, the frequency and duration of unsupported rearings (standing on the hind legs without any support) that is negatively related to anxiety was also recorded manually.

### The social interaction test

The test was conducted in the same open square box used for the open field. The box was separated into two “chambers” of equal dimensions (40 × 20 × 30 cm) connected with a smaller corridor. In each chamber a mesh-wire cage was placed near the wall opposite to the corridor (5 cm distance between the wall and the cage) in equal distance from the other two walls. Habituation was performed for 5 min in the set-up and 24 h later, an unknown mouse of the same sex as the tested animal was placed in one of the cages (counterbalanced between cohorts) and the test animal was placed again in the corridor of the set-up and left to explore the set-up for five minutes. The time spent in close proximity to the unknown con-specific as well as the time spent in close proximity to the empty cage have been determined. Animals not exploring the two cages for at least 10 sec have been excluded from any further analysis (3 males and 2 females, distributed in various dose-groups). A Discrimination Index was calculated according to the following formula: Time spent in close proximity to the cage with the unknown con-specific/Total time spent in close proximity to either cage.

### The novel object location (NOL) test

The test was conducted in the same square box used for the open field test (40 × 40 × 40 cm) according to Denninger *et al*.^66^.The time each mouse spent exploring each one of the two objects, the one in its original position and the one in a new position was recorded and the Discrimination Index was calculated according to the following formula: Time spent exploring the object in new position/Total time spent exploring both objects.

### Forced swimming stress (FSS)

Each mouse was subjected for 10 min to inescapable swimming in a glass beaker (24 cm in height; 15 cm in diameter) containing tap water (24 °C) to a depth of 17 cm. The duration of the following behaviors was measured: struggling (climbing towards the walls, having the body mostly in a vertical position and rippling the water with the front legs), swimming (moving more than one leg, with the body mostly in horizontal position) and floating (staying immobile or moving only one leg in order to remain at the surface).

### Analysis of gene expression

The procedure was conducted as previously described^26^. The samples from same-sex siblings that belonged to the same treatment group were pooled prior to mRNA extractions. The number of samples used for qPCR were as follows: In males, DMSO: 7 Basal/7 Beh. T, 0.5×: 7/4–6, 10×: 5–6/6, 100×: 5–6/6, 500×: 5–6/6–7. In females, DMSO: 5 Basal/7–8 Beh. T, 0.5×: 7/6, 10×: 5/5–6, 100×: 6–7/6–7, 500×: 6–7/7. Primers for the analyzed genes were either designed using publicly available software or selected from the PrimerBank database (Supplementary Table S8). Relative gene expression was calculated using the raw Ct data and *β-actin* as reference gene; an additional reference gene (G*apdh*) has been evaluated but the variability of its expression levels was greater than that of *β-actin*. For each gene analyzed, within each qPCR plate we included certain samples derived from DMSO exposed animals (Basal and Beh. tested), which served as internal controls in order to normalize gene expression data across qPCR runs.

### Hormonal determinations

The collected trunk blood samples from both Basal animals and animals following FSS were centrifuged at 5,000 g for 10 min and the serum was kept at −80 °C until use. Serum corticosterone levels were determined using the MP Biomedicals LLC (USA) RIA kit for small rodents (sensitivity 1.25 ng/ml, intra-assay variation 4.4%, inter assay variation 6.5%), according to the directions of the manufacturer. Serum ACTH levels were determined by a mouse ACTH-specific ELISA kit (Elabscience Biotechnology Inc. USA; sensitivity 9.38 pg/ml, detection range 15.63–1000 pg/ml, coefficient of variation <10%), following the kit protocol.

### Statistics

Behavioral data have been analyzed by generalized linear models (GLM), with the dose of N1 (treatment), sex and treatment × sex as predictor factors and the treatment (litter) as a build nested predictor factor. In the case of statistically significant dose effects, Bonferroni *post hoc* tests were used to determine specific group differences. For the analyses of qPCR and hormonal data, the data or samples from same-sex siblings that belonged to the same treatment group were pooled, so that the litter was the unit in the statistics. Normality tests and outliers were explored via SPSS 22.0. Three-way ANOVA for the effect of exposure to mixture N1, of behavioral testing and of sex was used, followed, when appropriate, by Dunnett’s post hoc test for the effect of mixture exposure compared to DMSO exposure. Females were classified as having ‘high estrogen levels’ (late methestrus and proestrus) or ‘low estrogen levels’ for the rest of the menstrual cycle phases and the effect of estrogen levels was included as a covariate. Significance was accepted for P values < 0.05.

Principal component analyses (PCA) were performed A. over the levels of struggling behavior in FSS and the gene expression and hormonal levels following FSS (Beh. tested animals) and B. over levels of mobility in the open field or social interaction (which have preceded FSS by some days) and the gene expression levels following FSS of those genes not affected by the exposure to behavioral testing, as we could not know when the observed alterations in the rest of the genes have appeared i.e. if they have preceded or not open field testing or social interaction. For these analyses, behavioral scores of siblings have been averaged. For the PCA the Direct Oblimean rotation method has been used with the maximum number of iterations for convergence set at 100 and the threshold cut-off for coefficient set at 0.4.

## Supplementary information

Supplementary Information.

## Data Availability

The datasets generated and/or analyzed during the current study are available from the corresponding author upon reasonable request. All data generated or analyzed during this study are included in this published article.
