# Supplementary material for: Long term transcriptional and behavioral effects in mice developmentally exposed to a mixture of endocrine disruptors associated with delayed human neurodevelopment
Source: Sci Rep. 2020 Jun 9;10:9367. doi: 10.1038/s41598-020-66379-x (PMC7283331; doi:10.1038/s41598-020-66379-x)
Supplement: Supplementary file 1 — Supplementary Information. [file 41598_2020_66379_MOESM1_ESM.pdf]

Long term transcriptional and behavioral effects in mice developmentally exposed to a mixture of endocrine disruptors associated with delayed human neurodevelopment.

Anastasia Repouskou[1‡], Anastasia-Konstantina Papadopoulou[1,2‡], Emily Panagiotidou[1,2], Panagiotis Trichas[2], Christian Lindh[3], Åke Bergman[4], Chris Gennings[5], Carl-Gustaf Bornehag[5,6], Joëlle Rüegg[7], Efthymia Kitraki[1#]*, Antonios Stamatakis[2#]*

[‡] equal contribution
# equal contribution
[1]Basic Sciences lab, Faculty of Dentistry, School of Health Sciences, National and Kapodistrian University of Athens (NKUA), Athens, Greece
[2]Biology-Biochemistry lab, Faculty of Nursing, School of Health Sciences, NKUA, Athens, Greece
[3]Division of Occupational and Environmental Medicine, Department of Laboratory Medicine, Lund University, Lund, Sweden
[4]Department of Environmental Science, Stockholm University, SE-106 91 Stockholm, Sweden
[5]Icahn School of Medicine at Mount Sinai, New York, NY, USA
[6]Karlstad University, Karlstad, Sweden
[7]Uppsala University, Evolutionary Biology Centre, Department of Organismal Biology, Norbyvägen 18A, 752 36 Uppsala

*Corresponding authors:
Antonios Stamatakis Faculty of Nursing, School of Health Sciences, National and Kapodistrian University of Athens, (NKUA), Papadiamantopoulou 123, 11527 Athens, Greece. astam@nurs.uoa.gr
Efthymia Kitraki Faculty of Dentistry, School of Health Sciences, NKUA, Thivon 2 Goudi, 11527 Athens, Greece. ekitraki@dent.uoa.gr

SUPPLEMENTAL FIGURES AND TABLES

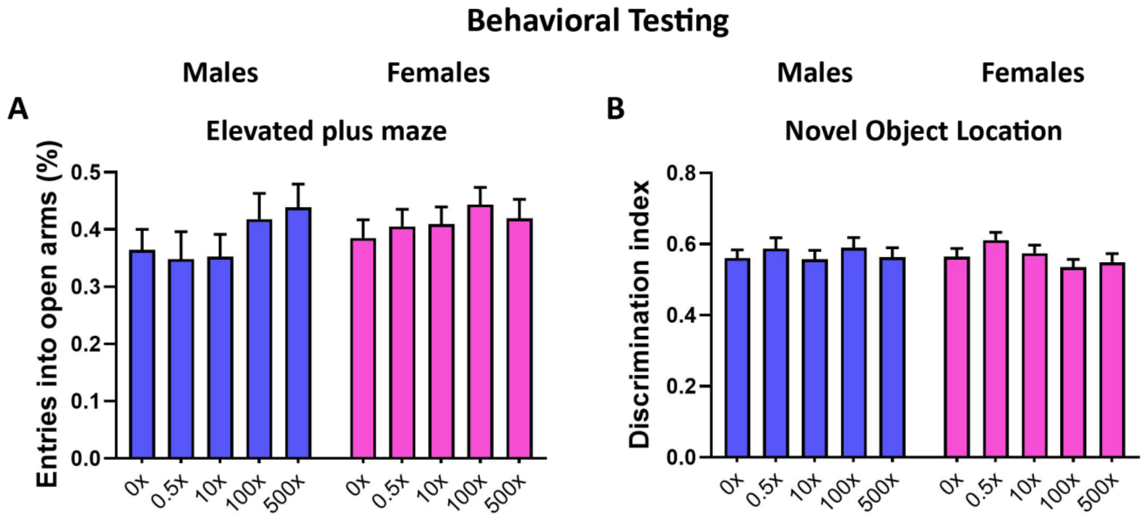

Supplementary Figure S1. Behavioral responses of adult male (blue bars) and female (magenda bars) mice *in utero* exposed to 0.5x, 10x, 100x and 500x SELMA mothers' levels of mixture N1 or the vehicle (0x) in the Elevated plus maze (A) and the Novel object location (B) tests. Bars represent the estimated marginal means ± SEM. Significance was accepted for P <0.05. No significant effects of mixture N1 exposure were detected.

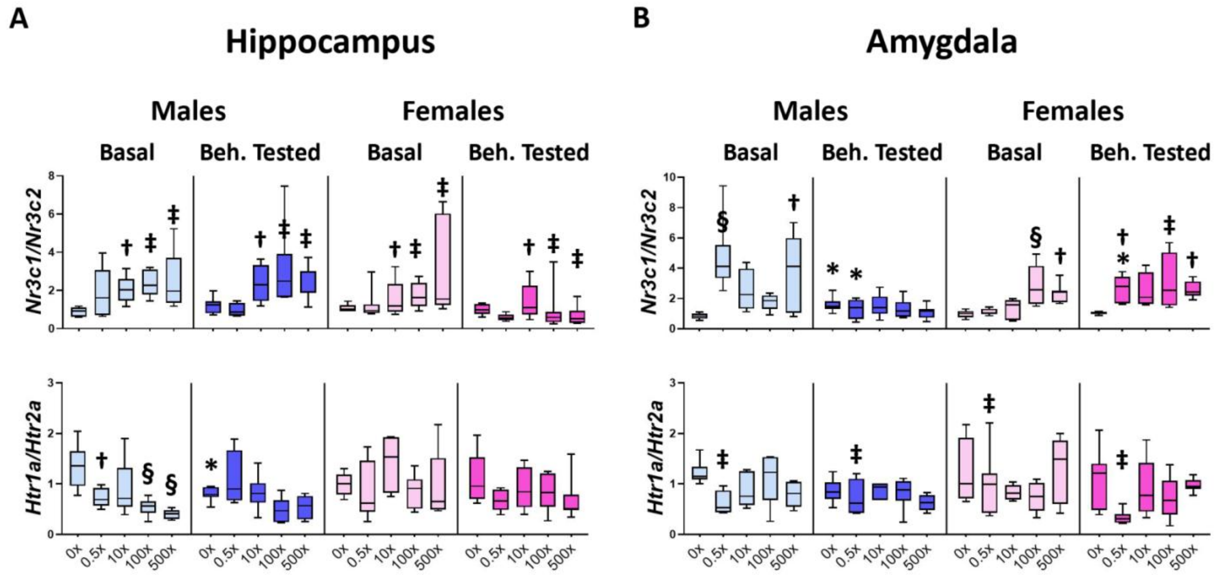

Supplementary Figure S2. Effect of mixture N1 on the *Nr3c1/ Nr3c2* and *Htr1a/ Htr2a* expression level ratios in (A) the hippocampus and (B) the amygdala of adult male (blue bars) and female (magenta bars) mice *in utero* exposed to 0.5x, 10x, 100x and 500x SELMA mothers' levels of mixture N1 or the vehicle (0x). Light colored bars show gene expression ratios under basal conditions (Basal) and darkly colored bars the gene expression ratios of matched siblings that underwent the behavioral tests (Beh. Tested). Expression levels of indicated genes were evaluated by qRT-PCR and normalized to *b-actin*. Box plots encompassing values from the 25th to 75th percentile of the data. The horizontal line in the box shows the median value, whereas the horizontal lines above and below the box show the maximal and minimal values, respectively. Significance was accepted for P <0.05. Significance is shown for the effect of mixture vs. the respective (Basal or Beh. Tested) control. † 0.01<P<0.05, ‡ 0.001<P<0.01, § P<0.001 as well as for the effect of Beh. Testing vs. the respective Basal group * P<0.05. Detailed statistics are provided in the Results section as well as in Supplemental tables S3, S3.1, S4 & S4.1.

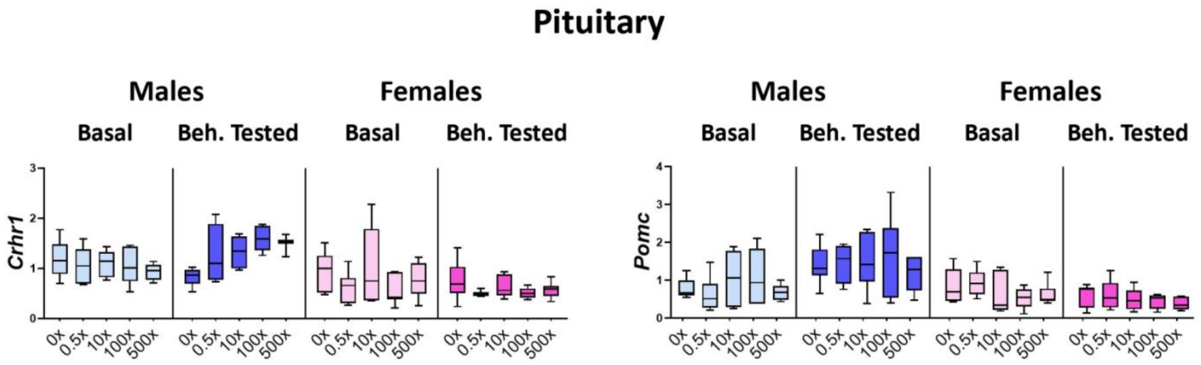

Supplementary Figure S3. Effect of mixture N1 on pituitary gene expression of adult male (blue bars) and female (magenta bars) mice *in utero* exposed to 0.5x, 10x, 100x and 500x SELMA mothers' levels of mixture N1 or the vehicle (0x). Light colored bars show gene expression under basal conditions (Basal) and darkly colored bars the gene expression of matched siblings that underwent the behavioral tests (Beh. Tested). Expression levels of indicated genes were evaluated by qRT-PCR and normalized to *b-actin*. Box plots encompassing values from the 25th to 75th percentile of the data. The horizontal line in the box shows the median value, whereas the horizontal lines above and below the box show the maximal and minimal values, respectively. Significance was accepted for P <0.05. No significant effects of mixture N1 exposure were detected.

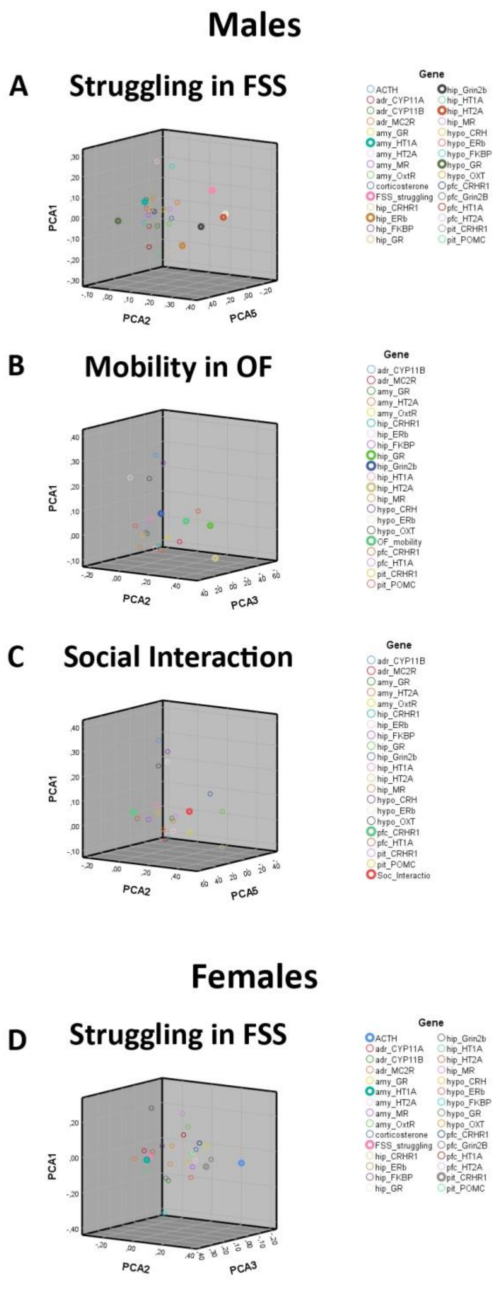

Supplementary Figure S4. Principal Component Analysis plots. Component plots in rotated space depicting Component Score Coefficients for levels of gene expression and behavioral scores for (A) struggling during Forced Swim Stress (FSS) of males, (B) mobility in open field (OF) of males, (C) social interaction of males and (D) struggling during Forced Swim Stress (FSS) of females. In each panel, the respective behavioral score and the genes clustered along with it are shown in bold circles.

Table S1. W values of the GLN and the respective Bonferroni post hoc comparisons between the DMSO-treated and the Mixture N1-treated groups are provided for all behaviors analyzed in this study (N/A: post hoc was not applicable). Significance was set for P < 0.05.

**Elevated plus maze MALES**	**GLN W/P values**	**0.5x**	**10x**	**100x**	**500x**
Entries into closed arms	5.811/0.214	N/A	N/A	N/A	N/A
Entries into open arms	5.241/0.263	N/A	N/A	N/A	N/A
% entries into open arms	2.239/0.692	N/A	N/A	N/A	N/A
Time in closed arms	2.125/0.713	N/A	N/A	N/A	N/A
Time in open arms	3.055/0.549	N/A	N/A	N/A	N/A
% time in open arms	2.324/0.676	N/A	N/A	N/A	N/A
**Elevated plus maze FEMALES**	**GLN W/P values**	**0.5x**	**10x**	**100x**	**500x**
Entries into closed arms	5.811/0.214	N/A	N/A	N/A	N/A
Entries into open arms	5.241/0.263	N/A	N/A	N/A	N/A
% entries into open arms	2.239/0.692	N/A	N/A	N/A	N/A
Time in closed arms	2.125/0.713	N/A	N/A	N/A	N/A
Time in open arms	3.055/0.549	N/A	N/A	N/A	N/A
% time in open arms	2.324/0.676	N/A	N/A	N/A	N/A

**Social Interaction** MALES	**GLN W/P values**	0.5x	10x	100x	500x
Discrimination Index	**28.576/<0.001**	**0.002**	1.000	1.000	**0.031**
**Social Interaction** FEMALES	**GLN W/P values**	0.5x	10x	100x	500x
Discrimination Index	1.861/0.761	N/A	N/A	N/A	N/A

T**able S1.** (continued)

**Open Field** MALES	**GLN W/P values**	0.5x	10x	100x	500x
Total distance moved	**20.485/<0.001**	1.000	**0.010**	**0.008**	**0.003**
Time in center	2.976/0.562	N/A	N/A	N/A	N/A
Frequency in center	7.681/0.104	N/A	N/A	N/A	N/A
Free rearings	1.260/0.868	N/A	N/A	N/A	N/A
Rearings against walls	1.256/0.869	N/A	N/A	N/A	N/A
**Open Field** FEMALES	**GLN W/P values**	0.5x	10x	100x	500x
Total distance moved	10.954/0.027	N/A	N/A	N/A	N/A
Time in center	2.976/0.562	N/A	N/A	N/A	N/A
Frequency in center	7.681/0.104	N/A	N/A	N/A	N/A
Free rearings	1.260/0.868	N/A	N/A	N/A	N/A
Rearings against walls	1.256/0.869	N/A	N/A	N/A	N/A

**Novel Object Location** MALES	**GLN W/P values**	0.5x	10x	100x	500x
Memory Index	4.172/0.383	N/A	N/A	N/A	N/A
**Novel Object Location** FEMALES	**GLN W/P values**	0.5x	10x	100x	500x
Memory Index	4.172/0.383	N/A	N/A	N/A	N/A

**Forced Swim Stress** MALES	**GLN W/P values**	0.5x	10x	100x	500x
Struggling	**21.321/<0.001**	0.686	**0.009**	**0.001**	**0.003**
Floating	2.282/0.684	N/A	N/A	N/A	N/A
**Forced Swim Stress** FEMALES	**GLN W/P values**	0.5x	10x	100x	500x
Struggling	**21.321/<0.001**	0.686	**0.009**	**0.001**	**0.003**
Floating	2.282/0.684	N/A	N/A	N/A	N/A

Table S2. Hypothalamus. F and P values of the ANOVAs for the effect of Mixture N1 and P values of the Dunnett's post hoc comparisons between the DMSO-treated and the Mixture N1-treated groups are provided for all genes analyzed in this study (B: Basal animals; BT: Behaviorally tested). N/A: post hoc was not applicable). Significance was set for P < 0.05.

Hypothalamus MALES	F (B / BT)<br>P (B / BT)	Post hoc<br>0.5 x (B / BT)	Post hoc<br>10x (B / BT)	Post hoc<br>100x (B / BT)	Post hoc<br>500x(B / BT)
Crh	4.645/4.645<br>**0.003/0.003**	0.51 / 0.51	0.98 / 0.98	**0.011** / **0.011**	0.66 / -0.66
Nr3c1	3.248/3.125<br>**0.031/0.033**	0.44 / 0.59	0.92 / 0.06	**0.040** / 0.16	0.46 / **0.010**
Fkbp5	1.695/10.774<br>0.184/ **<0.001**	N/A / 0.27	N/A / 0.19	N/A / **<0.001**	N/A / **<0.001**
Oxt	9.720/9.720<br>**<0.001 / <0.001**	0.99 / 0.99	0.81 / 0.81	**<0.001** / **<0.001**	**0.001** / **0.001**
Esr2	9.066/9.066<br>**<0.001** / **<0.001**	0.60 / 0.60	0.96 / 0.96	**<0.001** / **<0.001**	0.09/ 0.09
Hypothalamus FEMALES	F (B / BT)<br>P (B / BT)	Post hoc<br>0.5 x (B / BT)	Post hoc<br>10x (B / BT)	Post hoc<br>100x (B / BT)	Post hoc<br>500x(B / BT)
Crh	1.530/1.530<br>0.209/0.209	N/A	N/A	N/A	N/A
Nr3c1	1.949/0.514<br>0.134/0.726	N/A	N/A	N/A	N/A
Fkbp5	0.262/1.531<br>0.897/0.221	N/A	N/A	N/A	N/A
Oxt	0.637/0.637<br>0.638/0.638	N/A	N/A	N/A	N/A
Esr2	1.071/1.071<br>0.382/0.382	N/A	N/A	N/A	N/A

Table S2.1. Hypothalamus. F and P values of the ANOVAs for the effect of Mixture N1 and P values of the independent samples t- tests between the Basal and Behaviorally tested offspring per treatment group. F and P values refer to the triple interaction (treatment x behavioral testing x sex). N/A: the comparisons were not applicable. Significance was set for P < 0.05.

**Hypothalamus** MALES	**F / P values**	**DMSO** P values	**0.5x** P values	**10x** P values	**100x** P values	**500x** P values
Crh	1.766/0.143	N/A	N/A	N/A	N/A	N/A
Nr3c1	**2.897/0.026**	0.089	0.089	0.099	**0.011**	0.69
Fkbp5	**2.948/0.024**	**0.003**	**0.003**	0.806	0.177	0.109
Oxt	0.145/0.965	N/A	N/A	N/A	N/A	N/A
Esr2	0.993/0.416	N/A	N/A	N/A	N/A	N/A
**Hypothalamus** FEMALES	**F /P values**	**DMSO** P values	**0.5x** P values	**10x** P values	**100x** P values	**500x** P values
Crh	1.766/0.143	N/A	N/A	N/A	N/A	N/A
Nr3c1	**2.897/0.026**	0.194	0.221	0.581	0.069	0.634
Fkbp5	**2.948/0.024**	0.809	0.523	0.114	**0.003**	0.876
Oxt	0.145/0.965	N/A	N/A	N/A	N/A	N/A
Esr2	0.993/0.416	N/A	N/A	N/A	N/A	N/A

Table S3. Hippocampus. F and P values of the ANOVAs for the effect of Mixture N1 and P values of the Dunnett's post hoc comparisons between the DMSO-treated and the Mixture N1-treated groups are provided for all genes analyzed in this study (B: Basal animals; BT: Behaviorally tested). N/A: post hoc was not applicable). Significance was set for P < 0.05.

Hippocampus MALES	F (B / BT)<br>P (B / BT)	Post hoc<br>0.5 x (B / BT)	Post hoc<br>10x (B / BT)	Post hoc<br>100x (B / BT)	Post hoc<br>500x(B / BT)
Nr3c1	5.563/5.563<br>**0.001/ 0.001**	0.75 / 0.75	**0.002** / **0.002**	**0.001** / **0.001**	0.20 / 0.20
Nr3c2	3.771/3.246<br>**0.015/0.028**	0.48 / 0.20	**0.021** / 0.99	**0.038** / 0.42	**0.012** / 0.48
Nr3c1 / Nr3c2	5.424 / 5.424<br>**0.001 / 0.001**	0.961 / 0.961	**0.034 / 0.034**	**0.006 / 0.006**	**0.002 / 0.002**
Fkbp5	1.801<br>0.134	N/A	N/A	N/A	N/A
Esr2	0.871<br>0.484	N/A	N/A	N/A	N/A
Crhr1	9.125 /9.125<br>**<0.001/<0.001**	0.97 / 0.97	0.72 / 0.72	**0.028** / **0.028**	**0.001** / **0.001**
Htr1a	11.628 /9.147<br>**<0.001/<0.001**	**0.002** / 1.00	**0.001** / 0.25	**0.001** / **0.008**	**<0.001** / **0.024**
Htr2a	1.413/1.413<br>0.242/0.242	N/A	N/A	N/A	N/A
Htr1a / Htr2a	7.361/3.176<br>**<0.001/0.031**	**0.013** / 0.412	0.109 / 0.999	**0.001** / 0.221	**<0.001** / 0.385
Grin2b	3.246<br>**0.015**	0.40 / 0.40	0.40 / 0.40	0.08 / 0.08	0.85 / 0.85
Hippocampus FEMALES	F (B / BT)<br>P (B / BT)	Post hoc<br>0.5 x (B / BT)	Post hoc<br>10x (B / BT)	Post hoc<br>100x (B / BT)	Post hoc<br>500x(B / BT)
Nr3c1	0.233/0.233<br>0.919/0.919	N/A	N/A	N/A	N/A
Nr3c2	2.124/ 3.756<br>0.108 / **0.014**	N/A / 0.12	N/A / 0.40	N/A / 0.31	N/A / 0.97
Nr3c1 / Nr3c2	5.424 / 5.424<br>**0.001 / 0.001**	0.961 / 0.961	**0.034 / 0.034**	**0.006 / 0.006**	**0.002 / 0.002**
Fkbp5	1.801<br>0.134	N/A	N/A	N/A	N/A
Esr2	0.871<br>0.484	N/A	N/A	N/A	N/A
Crhr1	2.080/2.080<br>0.095/0.095	N/A	N/A	N/A	N/A
Htr1a	0.792/1.180<br>0.542/0.341	N/A	N/A	N/A	N/A
Htr2a	3.574/3.574	0.09 / 0.09	0.86 / 0.86	0.66 / 0.66	0.07 / 0.07

	**0.011/0.011**				
Htr1a / Htr2a	1.243/1.448<br>0.317/0.243	N/A	N/A	N/A	N/A
Grin2b	3.246<br>**0.015**	0.40 / 0.40	0.40 / 0.40	0.08 / 0.08	0.85 / 0.85

Table S3.1. Hippocampus. F and P values of the ANOVAs for the effect of Mixture N1 and P values of the independent samples t- tests between the Basal and Behaviorally tested offspring per treatment group. F and P values refer to the triple interaction (treatment x behavioral testing x sex). N/A: the comparisons were not applicable. Significance was set for P < 0.05.

**Hippocampus** MALES	**F / P values**	**DMSO** P values	**0.5x** P values	**10x** P values	**100x** P values	**500x** P values
Nr3c1	1.722/0.151	N/A	N/A	N/A	N/A	N/A
Nr3c2	**2.998/0.022**	0.763	0.119	**0.025**	0.512	0.313
Fkbp5	0.680/0.607	N/A	N/A	N/A	N/A	N/A
Esr2	2.301/0.064	N/A	N/A	N/A	N/A	N/A
Crhr1	2.388/0.056	N/A	N/A	N/A	N/A	N/A
Htr1a	**3.295/0.014**	0.081	0.058	**0.042**	**0.049**	0.180
Htr2a	0.495/0.740	N/A	N/A	N/A	N/A	N/A
Htr1a / Htr2a	**2.517/0.046**	**0.019**	0.175	0.837	0.648	0.148
Grin2b	1.108/0.357	N/A	N/A	N/A	N/A	N/A
**Hippocampus** FEMALES	**F /P values**	**DMSO** P values	**0.5x** P values	**10x** P values	**100x** P values	**500x** P values
Nr3c1	1.722/0.151	N/A	N/A	N/A	N/A	N/A
Nr3c2	**2.998/0.022**	0.956	0.131	**0.049**	**0.038**	0.195
Fkbp5	0.680/0.607	N/A	N/A	N/A	N/A	N/A
Esr2	2.301/0.064	N/A	N/A	N/A	N/A	N/A
Crhr1	2.388/0.056	N/A	N/A	N/A	N/A	N/A
Htr1a	**3.295/0.014**	0.693	0.872	0.135	0.08	0.127
Htr2a	0.495/0.740	N/A	N/A	N/A	N/A	N/A
Htr1a / Htr2a	**2.517/0.046**	0.639	0.487	0.118	0.953	0.358
Grin2b	1.108/0.357	N/A	N/A	N/A	N/A	N/A

Table S4. Amygdala. F and P values of the ANOVAs for the effect of Mixture N1 and P values of the Dunnett's post hoc comparisons between the DMSO-treated and the Mixture N1-treated groups are provided for all genes analyzed in this study (B: Basal animals; BT: Behaviorally tested). N/A: post hoc was not applicable). Significance was set for P < 0.05.

Amygdala<br>MALES	F (B / BT)<br>P (B / BT)	Post hoc<br>0.5 x (B / BT)	Post hoc<br>10x (B / BT)	Post hoc<br>100x (B / BT)	Post hoc<br>500x(B / BT)
Nr3c1	2.853/2.321<br>**0.044**/ 0.085	0.09 / 0.06	0.99 / 0.38	0.99 / 0.11	0.06 / 0.08
Nr3c2	17.575/ 0.255<br>**<0.001**/0.904	**<0.001**/N/A	**<0.001**/ N/A	**<0.001**/ N/A	**<0.001**/ N/A
Nr3c1 / Nr3c2	5.404/0.701<br>**0.003** / 0.599	**0.001** / N/A	0.215 / N/A	0.713/ N/A	**0.024** / N/A
Htr1a	3.503/2.023<br>**0.020** /0.122	**0.037** / N/A	**0.043** / N/A	1.00 / N/A	0.39 / N/A
Htr2a	2.154/4.922<br>0.104 /**0.005**	N/A / 1.00	N/A / 0.08	N/A / 0.84	N/A / 0.16
Htr1a / Htr2a	5.927 /5.927<br>**0.017 /0.017**	**0.002 / 0.002**	0.136 / 0.136	0.101/0.101	0.342 / 0.342
Oxtr	0.603/0.603<br>0.662/ 0.662	N/A	N/A	N/A	N/A
Amygdala<br>FEMALES	F (B / BT)<br>P (B / BT)	Post hoc<br>0.5 x (B / BT)	Post hoc<br>10x (B / BT)	Post hoc<br>100x (B / BT)	Post hoc<br>500x(B / BT)
Nr3c1	2.995/10.933<br>**0.039**/ **<0.001**	1.00 / **<0.001**	1.00 / 0.47	0.17 / **0.049**	0.08 / **<0.001**
Nr3c2	0.968/1.033<br>0.443/0.412	N/A	N/A	N/A	N/A
Nr3c1 / Nr3c2	7.600/4.612<br>**<0.001/0.007**	0.991 / **0.014**	0.882 / 0.066	**0.001** /**0.007**	**0.016** / **0.015**
Htr1a	2.572 /8.933<br>0.064/**<0.001**	N/A / 0.54	N/A / 0.96	N/A / 0.95	N/A /**0.001**
Htr2a	0.563/7.895<br>0.692 /**<0.001**	N/A / **0.001**	N/A / 0.83	N/A / 0.58	N/A / **0.001**
Htr1a / Htr2a	5.927 /5.927<br>**0.017 /0.017**	**0.002 / 0.002**	0.136 / 0.136	0.101/0.101	0.342 / 0.342
Oxtr	6.279/6.279<br>**<0.001/<0.001**	1.00/ 1.00	0.58 / 0.58	0.99 / 0.99	**<0.001/ <0.001**

Table S4.1. Amygdala. F and P values of the ANOVAs for the effect of Mixture N1 and P values of the independent samples t- tests between the Basal and Behaviorally tested offspring per treatment group. F and P values refer to the triple interaction (treatment x behavioral testing x sex). N/A: the comparisons were not applicable. Significance was set for P < 0.05.

**Amygdala**<br>MALES	**F / P values**	**DMSO**<br>P values	**0.5x**<br>P values	**10x**<br>P values	**100x**<br>P values	**500x**<br>P values
Nr3c1	**6.840/<0.001**	0.128	**0.002**	0.966	0.478	0.095
Nr3c2	**2.501/0.047**	**0.026**	**0.027**	**0.036**	0.183	0.064
Nr3c1/Nr3c2	**6.063/<0.001**	**0.004**	**0.029**	0.13	0.215	0.091
Htr1a	**2.976/0.023**	0.117	0.108	0.404	0.223	0.236
Htr2a	**3.407/0.012**	0.057	0.448	0.153	0.513	0.664
Oxtr	1.795/0.136	N/A	N/A	N/A	N/A	N/A
**Amygdala**<br>FEMALES	**F /P values**	**DMSO**<br>P values	**0.5x**<br>P values	**10x**<br>P values	**100x**<br>P values	**500x**<br>P values
Nr3c1	**6.840/<0.001**	0.698	**0.005**	0.114	0.529	0.051
Nr3c2	**2.501/0.047**	0.961	0.534	0.536	0.308	0.150
Nr3c1/Nr3c2	**6.063/<0.001**	0.5	**0.008**	0.1	0.867	0.432
Htr1a	**2.976/0.023**	0.145	**0.009**	0.343	0.877	**0.016**
Htr2a	**3.407/0.012**	0.313	0.053	0.062	0.845	**0.002**
Oxtr	1.795/0.136	N/A	N/A	N/A	N/A	N/A

Table S5. Prefrontal cortex. F and P values of the ANOVAs for the effect of Mixture N1 and P values of the Dunnett's post hoc comparisons between the DMSO-treated and the Mixture N1-treated groups are provided for all genes analyzed in this study (B: Basal animals; BT: Behaviorally tested). N/A: post hoc was not applicable). Significance was set for P < 0.05

Prefrontal MALES	F (B / BT)<br>P (B / BT)	Post hoc<br>0.5 x (B / BT)	Post hoc<br>10x (B / BT)	Post hoc<br>100x (B / BT)	Post hoc<br>500x(B / BT)
Crhr1	3.585/3.585<br>**0.012/ 0.012**	0.13 / 0.13	**0.023 / 0.023**	**0.011 / 0.011**	0.85 / 0.85
Grin2b	14.384 /1.523<br>**<0.001/**0.208	**<0.001**/ N/A	**<0.001**/ N/A	**<0.001**/ N/A	**<0.001**/ N/A
Htr1a	2.141/2.141<br>0.092/0.092	N/A	N/A	N/A	N/A
Htr2a	6.550/0.495<br>**<0.001/**0.739	**<0.001**/ N/A	**0.001**/ N/A	**<0.001**/ N/A	**0.003** / N/A
Htr1a/ Htr2a	1.140<br>0.342	N/A	N/A	N/A	N/A
Prefrontal FEMALES	F (B / BT)<br>P (B / BT)	Post hoc<br>0.5 x (B / BT)	Post hoc<br>10x (B / BT)	Post hoc<br>100x (B / BT)	Post hoc<br>500x(B / BT)
Crhr1	2.941/2.941<br>**0.028/0.028**	1.00 / 1.00	0.07 / 0.07	0.94 / 0.94	0.07 / 0.07
Grin2b	14.384 /1.523<br>**<0.001/**0.208	**<0.001**/ N/A	**<0.001**/ N/A	**<0.001**/ N/A	**<0.001**/ N/A
Htr1a	2.538/2.538<br>**0.049/0.049**	0.12 / 0.12	1.00 / 1.00	0.16 / 0.16	1.00 / 1.00
Htr2a	6.550/0.495<br>**<0.001**/0.739	**<0.001** / N/A	**0.001**/ N/A	**<0.001** / N/A	**0.003** / N/A
Htr1a/ Htr2a	1.140<br>0.342	N/A	N/A	N/A	N/A

Table S5.1. Prefrontal Cortex. F and P values of the ANOVAs for the effect of Mixture N1 and P values of the independent samples t- tests between the Basal and Behaviorally tested offspring per treatment group. F and P values refer to the interaction (treatment x behavioral testing) since no triple interaction (treatment x behavioral testing x sex) was statistically significant for the analyzed genes in this brain area. N/A: the comparisons were not applicable. Significance was set for P < 0.05.

**Prefrontal** BOTH SEXES	**F / P values**	**DMSO** P values	**0.5x** P values	**10x** P values	**100x** P values	**500x** P values
Crhr1	0.819/0.516	N/A	N/A	N/A	N/A	N/A
Grin2b	**2.961/0.023**	0.741	**0.008**	**0.002**	**0.001**	**0.003**
Htr1a	0.720/0.580	N/A	N/A	N/A	N/A	N/A
Htr2a	**2.583/0.042**	0.494	**< 0.001**	**0.001**	**0.001**	**0.002**
Htr1a/ Htr2a	1.259/ 0.291	N/A	N/A	N/A	N/A	N/A

Table S6. Adrenals. F and P values of the ANOVAs for the effect of Mixture N1 and P values of the Dunnett's post hoc comparisons between the DMSO-treated and the Mixture N1-treated groups are provided for all genes analyzed in this study (B: Basal animals; BT: Behaviorally tested). N/A: post hoc was not applicable). Significance was set for P < 0.05.

ADRENALS MALES	F (B / BT)<br>P (B / BT)	Post hoc<br>0.5 x (B / BT)	Post hoc<br>10x (B / BT)	Post hoc<br>100x (B / BT)	Post hoc<br>500x(B / BT)
Cyp11a1	5.559/ 6.921<br>**0.002/0.001**	**0.001** / 0.17	**0.023** / 0.14	**0.03 / <0.001**	0.08 / **0.029**
Cyp11b1	1.606/1.437<br>0.203/0.251	N/A	N/A	N/A	N/A
Mc2r	1.388/1.388<br>0.250	N/A	N/A	N/A	N/A
ADRENALS FEMALES	F (B / BT)<br>P (B / BT)	Post hoc<br>0.5 x (B / BT)	Post hoc<br>10x (B / BT)	Post hoc<br>100x (B / BT)	Post hoc<br>500x(B / BT)
Cyp11a1	5.005/1.506<br>**0.004**/ 0.227	0.95 / N/A	0.019*/ N/A<br>*at low E2	1.00 / N/A	1.00 / N/A
Cyp11b1	1.922/3.268<br>0.139 /**0.026**	N/A / **0.016**	N/A / 0.12	N/A / **0.019**	N/A /0.14
Mc2r	9.035/9.035<br>**<0.001/<0.001**	**0.048 / 0.048**	**0.040 / 0.040**	**<0.001/<0.001**	**<0.001/<0.001**

Table S6.1. Adrenals. F and P values of the ANOVAs for the effect of Mixture N1 and P values of the independent samples t- tests between the Basal and Behaviorally tested offspring per treatment group. F and P values refer to the triple interaction (treatment x behavioral testing x sex). N/A: the comparisons were not applicable. Significance was set for P < 0.05.

**Adrenals**<br>MALES	**F / P values**	**DMSO**<br>P values	**0.5x**<br>P values	**10x**<br>P values	**100x**<br>P values	**500x**<br>P values
Cyp11a1	**3.765/0.007**	**0.012**	**0.037**	**0.013**	0.926	0.167
Cyp11b1	**3.262/0.015**	0.217	0.371	0.448	0.227	0.868
Mc2r	0.200/0.938	N/A	N/A	N/A	N/A	N/A
**Adrenals**<br>FEMALES	**F /P values**	**DMSO**<br>P values	**0.5x**<br>P values	**10x**<br>P values	**100x**<br>P values	**500x**<br>P values
Cyp11a1	**3.765/0.007**	0.498	0.102	**0.032**	0.159	**0.029**
Cyp11b1	**3.262/0.015**	0.236	**0.026**	0.072	0.257	0.137
Mc2r	0.200/0.938	N/A	N/A	N/A	N/A	N/A

Table S7. Daily exposure of pregnant mice (µg / Kg BW) through food to Mixture N1 components. x refers to the geometric mean of SELMA mothers' levels for the chemicals in Mixture N1.

Mixture N1 components	0.5x	10x	100x	500x
**MEP**	0.4349	86.975	869.747	4348.734
**MBP**	0.4430	88.595	885.952	4429.760
**MBzP**	0.1016	20.325	203.254	1016.271
**MIDP/MPHP**	0.0886	17.713	177.132	885.658
**BPA**	0.0075	1.500	14.999	74.994
**TCP**	0.0058	1.154	11.541	57.703
**3-PBA**	0.0016	0.329	3.294	16.469
**p, p'-DDE**	0.0131	2.623	26.226	131.129

Table S8. List of primers used in qRT-PCR

Gene	*Forward primer (5'-3')*	*Reverse primer (5'-3')*	T annealing	*Primer Bank ID / Reference*
Mc2r	CATCTTGCCGAGAAAGATCCTA	CCTTGGCTTTGTCACTAATGCT	58°C	Ref.1
Cyp11a1	AGGTCCTTCAATGAGATCCCTT	TCCCTGTAAATGGGGCCATAC	58°C	9789921a1
Cyp11b1	GATACAGATCCTGAGGGAGC	CCGGCAACGTCACAAACACA	59°C	Ref. 2
Pomc	TAGATGTGTGGAGCTGGTGC	CGTACTTCCGGGGGTTTTCA	58°C	This study
Crhr1	GGAACCTCATCTCGGCTTTCA	GTTACGTGGAAGTAGTTGTAGGC	59°C	6681013a1
Crh	GAGGCATCCTGAGAGAAGTCC	GTTAGGGGCGCTCTCTTCTC	60°C	This study
Nr3c1	GGACCACCTCCCAAACTCTG	GCTGTCCTTCCACTGCTCTT	60°C	This study
Nr3c2	GAAAGGCGCTGGAGTCAAGT	TGTTCGGAGTAGCACCGGAA	60°C	17384009a1
Fkbp5	TGAGGGCACCAGTAACAATGG	CAACATCCCTTTGTAGTGGACAT	60°C	Ref. 3
Esr2	ACACCTTGCCTGTAAACAGAGA	GCAGAAGTGAGCATCCCTCTT	58°C	46877093c3
Htr1a	GGATGTTTTCCTGTCCTGGT	CACAAGGCCTTTCCAGAACT	59°C	Ref.4
Htr2a	AGAACCCCATTCACCATAGC	ATCCTGTAGCCCGAAGACTG	59°C	Ref. 4
Grin2b	TGGCCCTCAGCCTCATCACC	CATCACGGATTGGCGCTCCT	60°C	Ref. 5
Oxtr	GCACGGGTCAGTAGTGTCAA	AAGCTTCTTTGGGCGCATTG	59°C	This study
Oxt	CACCTACAGCGGATCTCAGAC	CCGAGGTCAGAGCCAGTAAG	60°C	This study
β-actin	GGCTGTATTCCCCTCCATCG	CCAGTTGGTAACAATGCCATGT	59°C	6671509a1

References

1. Chida D, et al. Melanocortin 2 receptor is required for adrenal gland development, steroidogenesis, and neonatal gluconeogenesis. Proc Natl Acad Sci U S A. 104(46):18205-10, 2007.

2. Kinyua AW, et al. Insulin Regulates Adrenal Steroidogenesis by Stabilizing SF-1 Activity. Sci Rep. Mar 22;8(1):5025, 2018.

3. Kitraki et al, Developmental exposure to bisphenol A alters expression and DNA methylation of Fkbp5, an important regulator of the stress response. Mol. Cell. Endocrinol. 417, 191-199; 2015.

4. Chiavegatto S, et al. Individual vulnerability to escalated aggressive behavior by a low dose of alcohol: decreased serotonin receptor mRNA in the prefrontal cortex of male mice. Genes Brain Behav. Feb;9(1):110-9, 2010.

5. Masocha W. Astrocyte activation in the anterior cingulate cortex and altered glutamatergic gene expression during paclitaxel-induced neuropathic pain in mice. PeerJ. Oct 22;3:e1350, 2015.
